# Effects of high-intensity interval training on physical morphology, cardiopulmonary function, and metabolic indicators in older adults: a systematic review and meta-analysis

**DOI:** 10.3389/fendo.2025.1526991

**Published:** 2025-03-25

**Authors:** Jie Men, Chengrui Zhao, Chenmin Xiang, Guoyu Zhu, Zhengyang Yu, Pengbo Wang, Simin Wu, Yuxi Zhang, Yishan Li, Liuliu Wang, Xueyan Gong, Xiang Yang, Shuangling Zou, Jia Ma, Chenglong Cui, Hao Li, Xuedi Ma, Wenjie Wu, Yaoming Wang

**Affiliations:** ^1^ Fenyang College, Shanxi Medical University, Fenyang, China; ^2^ Third Hospital of Shanxi Medical University, Shanxi Bethune Hospital Department of Immunology & Rheumatology, Shanxi Academy of Medical Sciences, Tongji Shanxi Hospital, Taiyuan, Shanxi, China

**Keywords:** high-intensity interval training, older adults, physical morphology, cardiopulmonary function, metabolic indicators

## Abstract

**Background:**

Despite the growing attention towards the efficacy of high-intensity interval training (HIIT) on older adult health, a consensus regarding the pleiotropic effects of HIIT in this population is yet to be reached. Previous studies have predominantly focused on specific outcomes or particular groups, lacking comprehensive analysis.

**Objective:**

We aimed to conduct a systematic evaluation of the impact of HIIT on body composition, cardiopulmonary function, and metabolic parameters in older adults.

**Methods:**

The databases searched included PubMed, Web of Science, Cochrane Library, Scopus, WanFang, and other relevant sources from the inception of the database until July 2023. Randomized controlled trials (RCTs) on the effects of HIIT on body shape, cardiopulmonary function, and metabolic parameters in the older adult were searched.

**Results:**

A total of 87 RCTs meeting the criteria were included, involving 4,213 older adult people. Meta-analysis results showed that HIIT significantly improved body fat percentage (BF%) [MD: −1.63%, *p* = 0.005], maximal oxygen uptake (VO_2max_) [MD: 2.46 mL min^−1^ kg^−1^, *p* < 0.00001], maximal heart rate (HR_max_) [MD: 2.83 beats min^−1^, *p* = 0.02], and high-density lipoprotein (HDL) levels [MD: 0.04 mmol L^−1^, *p* = 0.002]. However, for systolic blood pressure (SBP) [MD: 0.49 mmHg, p = 0.60], resting heart rate (HR_rest_) [MD: −0.95 BPM ^−1^, p = 0.24], triglycerides (TG) [tendency for MD: −0.02 mmol L^−1^, p = 0.61], low-density lipoprotein (LDL) [MD: −0.04 mmol L^−1^, p = 0.27] had no significant effect. Sensitivity analysis found that HIIT significantly improved waist circumference (WC) [MD: −1.89 cm, *p* = 0.17], diastolic blood pressure (DBP) [MD: −0.63 mmHg, *p* = 0.23], respiratory exchange rate (RER) [MD: 0.01, *p* = 0.20], total cholesterol (TC) [MD: 0.10 mmol L^−1^, *p* = 0.14], and fasting plasma glucose (FPG) [MD:−0.20 mmol L^−1^, *p* = 0.08], but the results lacked robustness. There was no significant improvement in DBP [MD: −0.63 mmHg, *p* = 0.23] and body mass index (BMI) [MD: −0.36 kg m^−2^, *p* = 0.06].

**Conclusions:**

HIIT has shown certain potential and advantages in improving the physical health of the older adult, especially in cardiopulmonary function. However, more high-quality studies are needed to confirm the effects of HIIT on the physical health of the older adult in the future. It also provides a reference for the clinical practice and family health management of HIIT in the older adult and the development of HIIT guidelines.

**Systematic review registration:**

https://www.crd.york.ac.uk/PROSPERO/myprospero, identifier CRD42023460252.

## 1 Introduction

The latest research predicts that the global older adult population aged 60 and above is projected to reach 2 billion by 2050 (1). With advancing age, there is a decline in physiological system functionality and an increased susceptibility to stress response (2). Older adults experience rapid deterioration in functional capacity in response to acute illness or major events (3–5). At the same time, inadequate physical activity can accelerate the aging process and contribute to adverse outcomes, such as an increased susceptibility to falls, hospitalization, premature mortality, and all-cause mortality (6); compared with safe physical activity, unsafe physical activity will also increase the same risk. Therefore, the older adult should observe strict risk assessment and standardized guidance when engaging in physical activity (7, 8). It is important to note that while the decline in systemic function among older individuals can be prevented and delayed, it may become irreversible once it manifests as an adverse event. Therefore, in addition to mitigating risk factors for adverse events, greater emphasis should be placed on preventing/delaying the deterioration of systemic functions to achieve healthy aging (9). Copenhagen Consensus Statement 2019 highlights that physical activity is a crucial determinant for maintaining health and the normal functioning of physiological systems (6), and it represents one of the primary strategies to delay age-related decline in physical function among older adults (10). Despite its significant role in reducing mortality risk (11), cardiovascular disease (CVD), and certain cancers (12), a majority of older adult individuals refrain from engaging in exercise or leisure physical activities (13), owing to concerns regarding potential risks associated with exercise. A cross-sectional population study noted that the annual incidence of exercise-related cardiac arrest in the older adult is extremely rare (14), which means that the benefits of exercise far outweigh the possible risks.

An increasing number of studies have demonstrated that exercise can enhance age-related body composition (15); ameliorate dyslipidemia; improve cardiopulmonary function (16); prevent the onset of type 2 diabetes, hypertension, CVD, and cancer (17); and reduce overall mortality rates (18). The health benefits of exercise have garnered consensus among experts (12, 19). Currently, three forms of physical activity exhibit significant potential in promoting the health of older adults. The first category encompasses non-exercise physical activities (low intensity), such as walking and household chores. Recent evidence unequivocally indicates that low-intensity physical activities also confer health advantages for older adults, including reduced all-cause mortality and protection against Alzheimer’s disease (20, 21). The second form is moderate-intensity aerobic exercise, which is supported by substantial evidence establishing a dose–response relationship with health outcomes and recommended by global guidelines (12). The pleasure brought by exercise is positively correlated with compliance, but has an inverted U-shaped curve with exercise time (22), which is the main reason that the expected compliance rate of aerobic exercise is 27% (23), but the actual compliance rate is less than 5% (24). It is also the main reason for exercise paradox (beneficial but difficult to promote) (25). High-intensity interval training (HIIT) presents itself as a promising alternative for promoting health among older adults. Intermittent training incorporates short bursts of high-intensity anaerobic exercise followed by low-intensity aerobic recovery, yielding comparable exercise effects to moderate-intensity aerobic exercise. Moreover, HIIT offers advantages in terms of time efficiency and cost-effectiveness (26). It holds great potential for widespread utilization in older adult health promotion endeavors. Importantly, unlike other pharmacological treatments that solely target a single outcome, exercise typically exerts positive effects on multiple physiological systems (27). Recent studies have demonstrated that engaging in 3 to 4 min of HIIT per day can significantly reduce the risk of all-cause and cancer mortality by 38% to 40%, as well as decrease the risk of CVD mortality by 48% to 49% (28). Furthermore, numerous meta-analyses have provided support for the role of HIIT in enhancing the health status of older individuals (26, 29–37). However, previous investigations on the health benefits of HIIT in older adults possess certain limitations. Firstly, most outcome measures were assessed within a single (26, 30, 31, 34, 36) or dual (29, 32, 33) physiological system. Secondly, only specific diseases (26, 29, 31) or particular risk factors (32, 33, 35, 36) were considered. Thirdly, the majority of studies had small sample sizes and included non-older adult participants within their populations (26). Additionally, a meta-analysis encompassing 10 studies incorporated 6 studies from a single research group (36). These limitations inevitably weaken the advantages of HIIT, limit the comprehensive interpretation of the health benefits of HIIT in older adults, and lead to limitations in generalizability. Furthermore, a significant number of included studies were deemed to be of low quality (29, 30, 32). While most research findings support the positive health effects of HIIT on older adult individuals, particularly about certain physiological systems, it is important to note that these systematic reviews or meta-analyses primarily focus on specific improvements within individual physiological systems or diseases. Considering that the human body functions as an integrated whole, our emphasis lies in examining the overall health impacts of HIIT and recognizing the essentiality of valid scientific evidence when formulating strategies to address global health issues among older populations.

Given the above considerations, a systematic review was conducted to evaluate the effects of HIIT on 14 outcomes of body shape, cardiopulmonary function, and metabolism in older adults. We also conducted subgroup analysis on the factors that may affect the conclusions, such as study type, intervention period, and disease type, in order to obtain more comprehensive data and provide evidence support for clinical or family health management and the development of HIIT guidelines/recommendations.

## 2 Methods

### 2.1 Search strategy

The present review adhered to the guidelines of the Preferred Reporting Items for Systematic Reviews and Meta-analyses (PRISMA) (38). It was prospectively registered in the International Registry of Prospective Systematic Reviews (PROSPERO) database under registration number “CRD42023460252” on 5 September 2023. The following databases were electronically searched: PubMed, Web of Science, Cochrane Library, Scopus, and WanFang database. A comprehensive search was conducted for randomized controlled trials (RCTs) investigating the HIIT on body composition, cardiopulmonary function, and metabolic indicators in older adults. The search period ranged from the inception of each database to 1 July 2023. The search strategy is structured to include terms related to “aged,” “older adult,” “older,” “High-Intensity Interval Training,” “HIIT,” and “High-Intensity Intermittent Exercise.” Additionally, relevant literature and previously published systematic reviews were manually screened to identify any studies missed during the initial search process (search details in **Additional File 1** in **Supplementary Material**).

### 2.2 Inclusion criteria

**Figure 1:**
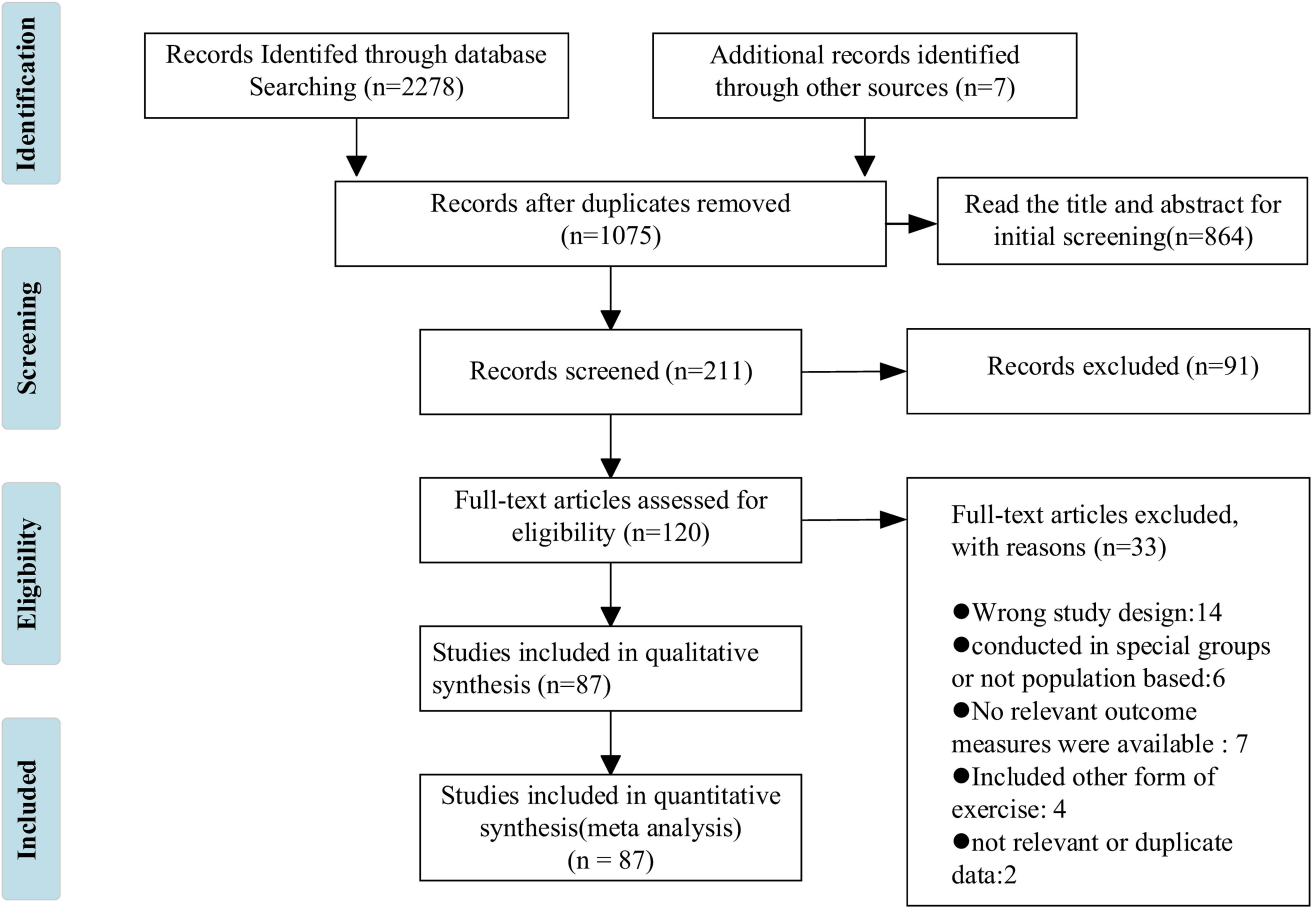
Study selection. The number of documents retrieved from the database is as follows: PubMed (*n* = 40), Embase (*n* = 694), Cochrane Library (*n* = 801), CNKI (*n* = 3), Scopus (*n* = 738), and WanFang Data (*n* = 2).

The retrieved literature was imported into NoteExpress, a literature management software. Two researchers independently conducted a comprehensive literature review, eliminating duplicate and irrelevant studies, extracting relevant data, and cross-verifying the selected literature. In case of any discrepancies, they consulted with each other or sought input from a third party for discussion. A screening flowchart is presented in **Figure 1**. The research object of this paper is the older adult, and subjects were not restricted by country, race, or sex, or physical condition, except athletes. The experimental group was HIIT, and the control group was blank control or low-intensity exercise control. Articles in Chinese or English reporting at least one outcome measure were also included. The outcome measures included body mass index (BMI), body fat percentage (BF%), waist circumference (WC), maximal oxygen uptake (VO_2max_), systolic blood pressure (SBP), diastolic blood pressure (DBP), resting heart rate (HR_rest_), maximal heart rate (HR_max_), respiratory exchange rate (RER), total cholesterol (TC), triglycerides (TG), high-density lipoprotein (HDL), low-density lipoprotein (LDL), and fasting plasma glucose (FPG).

### 2.3 Data extraction

The data extraction encompassed the following components: basic information of the included literature (first author’s name, title, and publication year), characteristics of study subjects (age, gender, and number of subjects), training variables (intensity, form, frequency, and intervention period), main results, and key elements of risk of bias assessment. To calculate effect sizes for physical fitness measures in the intervention and control groups, baseline and follow-up mean as well as standard deviations (SDs) were extracted. In case any required data were missing, we contacted the corresponding author to obtain them. If relevant data could not be provided by the author, they were excluded from the analysis. The characteristics of the included studies are presented in **Supplementary Table 1** (in **Additional File 2**).

### 2.4 Biased risk assessment

RCTs were analyzed using the Cochrane Risk of Bias Tool 2.0 (39). There are three levels: low risk, high risk, and uncertain. Two researchers used ReviewManager 5.4.1 software to rigorously evaluate seven aspects of randomized allocation methods, allocation concealment of randomized methods, blinding of research subjects and interveners, blinding of outcome evaluators, integrity of outcome data, possibility of selective reporting, and other sources of bias. The risk of biased judgment in each domain was interpreted as low risk, moderate risk, severe risk, borderline risk, or no information (40). Two reviewers independently assessed the risk of bias, and any disagreements were resolved through a third party. In addition, the risk of publication bias was assessed using funnel plots when the meta-analysis included ≥10 studies.

### 2.5 Statistical analysis

Review Manager 5.3 (RevMan) software was used for statistical analysis. Because the literature had the same continuous outcome variable and the same unit of measurement, mean differences (MDs) and SDs along with 95% confidence intervals (95% CIs) were used to estimate the effect sizes observed in the studies. The random-effects model (overall effect *p*-value < 0.05) and fixed-effects model (overall effect *p*-value > 0.05 under the random-effects model) were the two main models used to synthesize all the results of forest plots. *I* ^2^ was used to evaluate the degree of heterogeneity: above 25%, 50%, and 75% were low, medium, and high heterogeneity, respectively (41), and the level of meta-analysis was set as α = 0.05. When *I* ^2^ ≤ 25%, the fixed-effects model was used for combined analysis. If *I* ^2^ > 25%, the random-effects model was used for combined analysis. In order to increase the stability of the results, sensitivity analysis was performed by one-by-one elimination. These tests focused on significant heterogeneity associated with HIIT, in which individual studies were systematically removed from the meta-analysis and pooled effect estimates were recalculated to assess the impact of individual studies on the meta-analysis. As the objective of this study was to investigate the effects of HIIT on body shape, cardiopulmonary function, and metabolic parameters in the older adult, subgroup analyses were conducted according to intervention duration (≤12 weeks and >12 weeks) and disease type (CVD, diabetes, hypertension, cancer, and others) when *I* ^2^ ≥ 50%. The possible publication bias was evaluated by drawing a funnel plot. Further analysis was performed when more than five studies were included in the subgroup.

## 3 Results

### 3.1 Study selection

**Figure 2:**
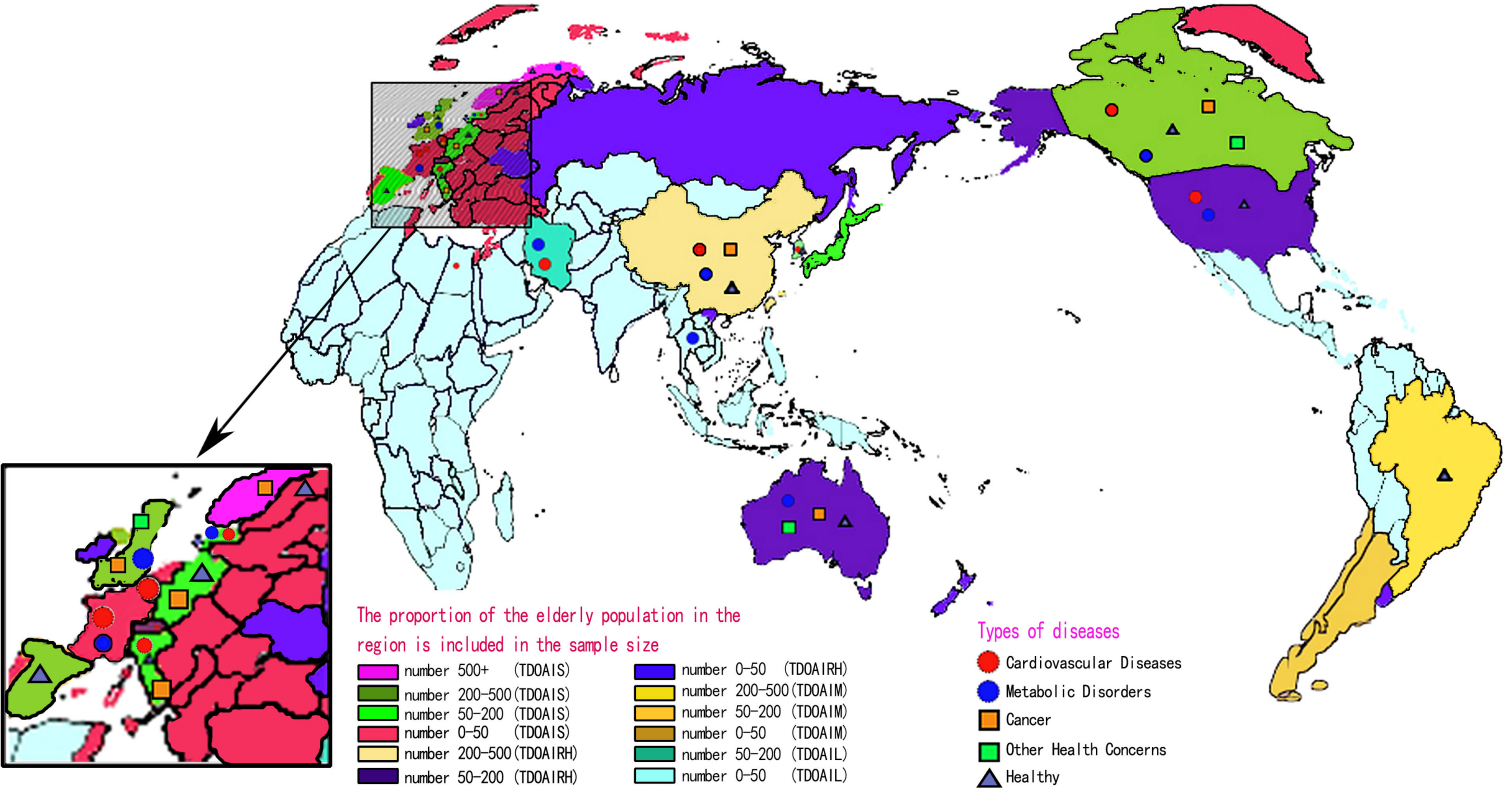
Contributing data source across world regions. TDOAIS, the degree of aging is serious; TDOAIRH, the degree of aging is relatively heavy; TDOAIM, the degree of aging is moderate; TDOAIL, the degree of aging is low. When the disease mark appears in a certain area, it means that the subjects included in the area have the disease or are in a healthy state. The proportion of the older adult population: TDOAIS > 17.91, 17.91 > TDOAIRH > 13.15, 13.15 > TDOAIM > 9.02, and TDOAIL < 9.02.

**Figure 1** describes the PRISMA process in detail. A total of 2,278 studies were retrieved from the database, and 7 studies were obtained from other sources. After the strict screening, 87 (42–128) studies were finally included, with a total of 4,213 older adult people. There were 2,099 subjects in the HIIT group, aged 66.8 ± 18.7 years, and 2,114 subjects in the control group, aged 63.1 ± 20 years. The exercise frequency ranged from 1 to 21 times per week, the duration ranged from 7 to 180 min per time, and the cycle ranged from 2 to 48 weeks. The measurement methods of outcome indicators were also reported. Among the included studies, 25 studies described medical supervision in detail, 29 did not describe it in detail, and 33 did not describe it. In addition, 24 studies reported a total of 196 people withdrawing due to family, subjective will, and other factors, accounting for 4.65% of the total number. There were 46 cases of adverse events, and the incidence of adverse events was 1.09%. Of note, adverse events were not attributed to HIIT. **Figure 2** shows the geographical and sample distribution of the included studies, with 65.16% of the studies in Europe, 14.62% in North America and South America,14.24% in Asia, 5.03% in Australia, and 0.95% in Africa, involving 21 countries and regions. Of these, 658 were from Norway, 543 were from Switzerland, 537 were from Belgium, 191 were from Denmark, 182 were from Spain, 200 were from the United Kingdom, 148 were from Sweden, 144 were from Germany, 116 were from Italy, 26 were from France, 244 were from Canada, 222 were from the United States, 150 were from Brazil, 254 were from China, 133 were from Iran, 88 were from Japan, 72 were from South Korea, 29 were from Thailand, 24 were from Indonesia, 212 were from Australia, and 40 were from Egypt, for a total of 4,213 individuals.

### 3.2 Quality assessment of included studies

A total of 87 RCTs were included, and the overall risk of bias was low. Of these RCTs, 84/87 evaluated using randomized sequence generation, 84/87 assessments used allocation concealment, 77/87 subjects and staff were blinded, 77/87 were blinded to the outcome data evaluation, 84/87 had complete data, 86/87 reported no selectivity, and 39/87 had no other bias among the included (the results are shown in **Additional File 3** in **Supplementary Material**).

### 3.3 Meta-analysis result

The studies on the physical effects of the older adult can be divided into three aspects: body shape, cardiopulmonary function, and metabolic function. A total of 14 indicators were involved, and the detailed results of all indicators are recorded in **Table 1**.

**Table 1:** Meta-analysis results.

Outcomes	The number of studies	*p*	*I*² (%)	Effect model	95%CI
BMI	27	0.06	70	RE	−0.36 [−0.72, 0.01]
BF%	16	0.005**	31	RE	−1.63 [−2.78, −0.48]
WC	16	0.17	80	RE	−1.89 [−4.62, 0.83]
VO_2max_	56	<0.00001**	79	RE	2.46 [1.73, 3.20]
SBP	38	0.60	77	RE	−0.49 [−2.29, 1.31]
DBP	37	0.23	77	RE	−0.63 [−1.68, 0.41]
HR_rest_	29	0.24	83	RE	−0.95 [−2.54,0.63]
HR_max_	39	0.02*	79	RE	2.83 [0.44,5.22]
RER	26	0.20	45	RE	0.01 [−0.00,0.02]
TC	20	0.14	34	RE	0.10 [−0.03, 0.23]
TG	26	0.61	36	RE	−0.02 [−0.10, 0.06]
HDL	25	0.002**	21	FE	0.04 [0.02, 0.07]
LDL	22	0.27	11	FE	−0.04 [−0.11, 0.03]
FPG	23	0.08	60	RE	−0.20 [−0.42, 0.03]

RE, random-effects model; FE, fixed-effects model; MD, mean difference; CI, confidence interval. *p<0.05.

#### 3.3.1 Body morphology indicators

**Figure 3:**
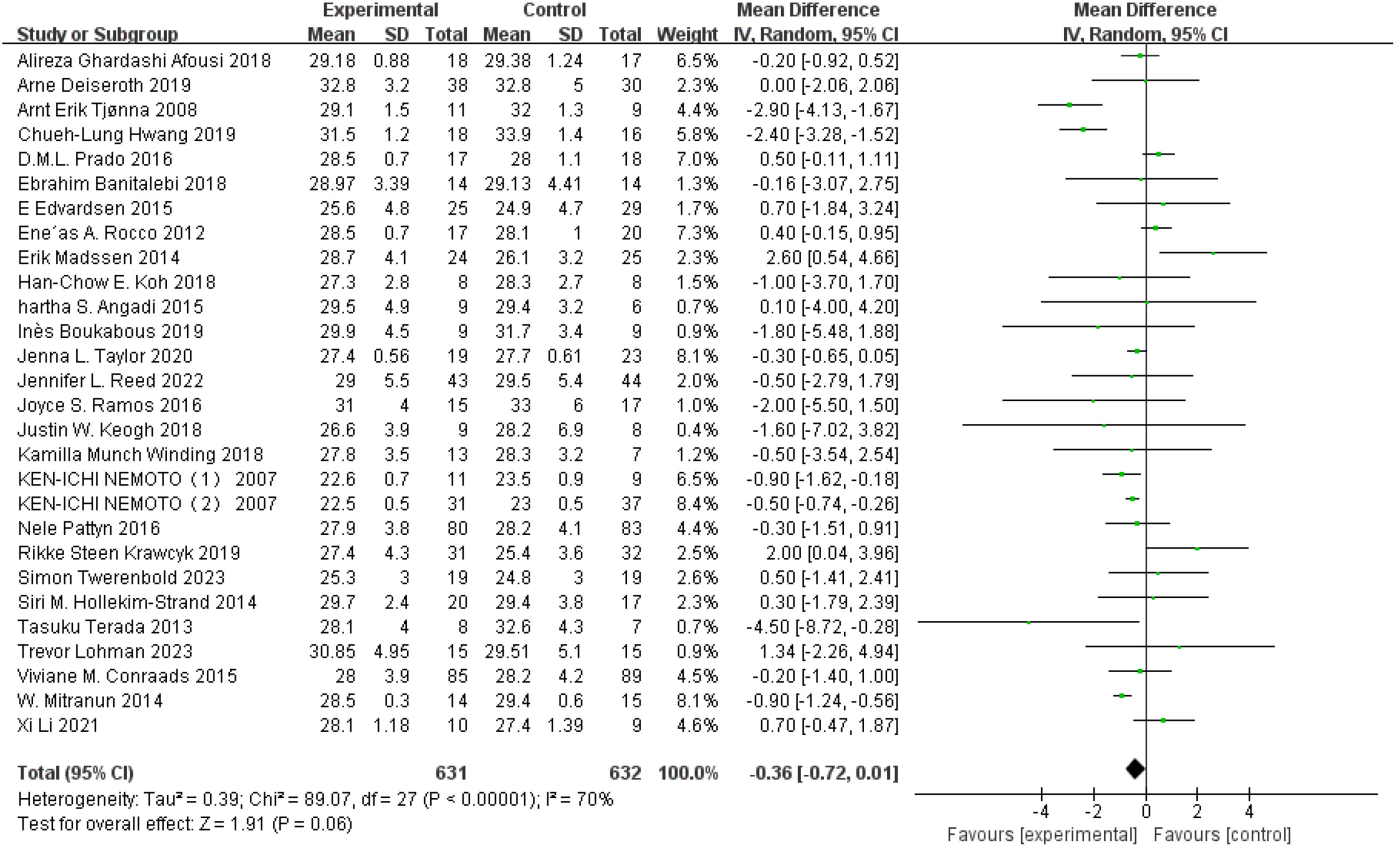
Forest plot of BMI.

BMI: A total of 27 (42, 43, 47, 50, 52, 53, 60, 67, 70–72, 75, 77–80, 84, 85, 88, 91, 100, 108, 109, 116, 123, 126, 127) studies were included. Compared with the control group, HIIT did not significantly improve BMI (MD: −0.36 kg m^−2^, *p* = 0.06) in the older adult, with statistical heterogeneity (*I* ^2^ = 70%) (**Figure 3**). Sensitivity analysis showed that the results of this study were robust. Funnel plots were basically symmetric, and there was no evidence of publication bias.

**Figure 4:**
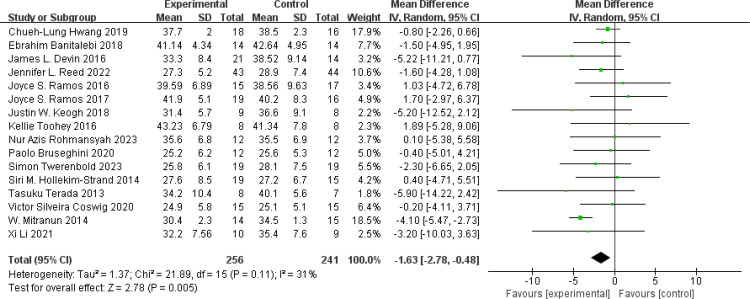
Forest plot of BF%.

BF%: A total of 16 (47, 50, 60, 73, 81, 87, 92, 103–105, 108, 111, 116, 118, 122, 123) studies were included. Compared with the control group, HIIT significantly improved BF% (MD: −1.63%, *p* = 0.05) without significant heterogeneity (*I* ^2^ = 31%) (**Figure 4**). Sensitivity analysis showed that the results of this study were robust. Funnel plots were basically symmetric, and there was no evidence of publication bias.

**Figure 5:**
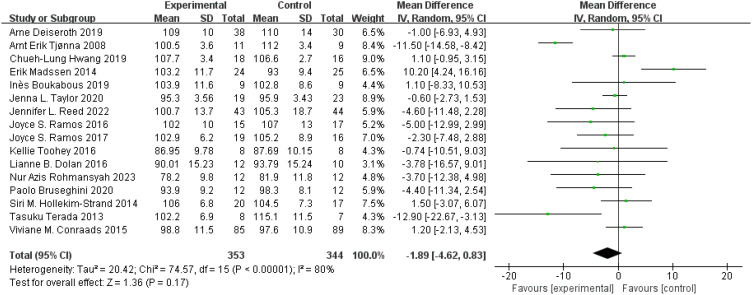
Forest plot of WC.

WC: A total of 16 (49, 50, 54, 58, 61, 73, 91, 103–105, 108, 111, 115–118) studies were included. Compared with the control group, HIIT had no significant effect on WC in the older adult (MD: −1.89 cm, *p* = 0.17), but there was statistical heterogeneity (*I* ^2^ = 80%) (**Figure 5**). After excluding the study by Tjønna et al. (117), *I* ^2^ decreased to 48%, which was not statistically significant (MD: −0.53 cm, *p* = 0.58). After excluding the study by Madssen et al. (91), there was no significant change in *I* ^2^, but HIIT significantly improved WC in the older adult (MD: −2.69 cm, *p* = 0.04). Sensitivity analysis suggested that the results were not robust. Funnel plots were basically symmetric, and there was no evidence of publication.

#### 3.3.2 Cardiopulmonary function indicators

**Figure 6:**
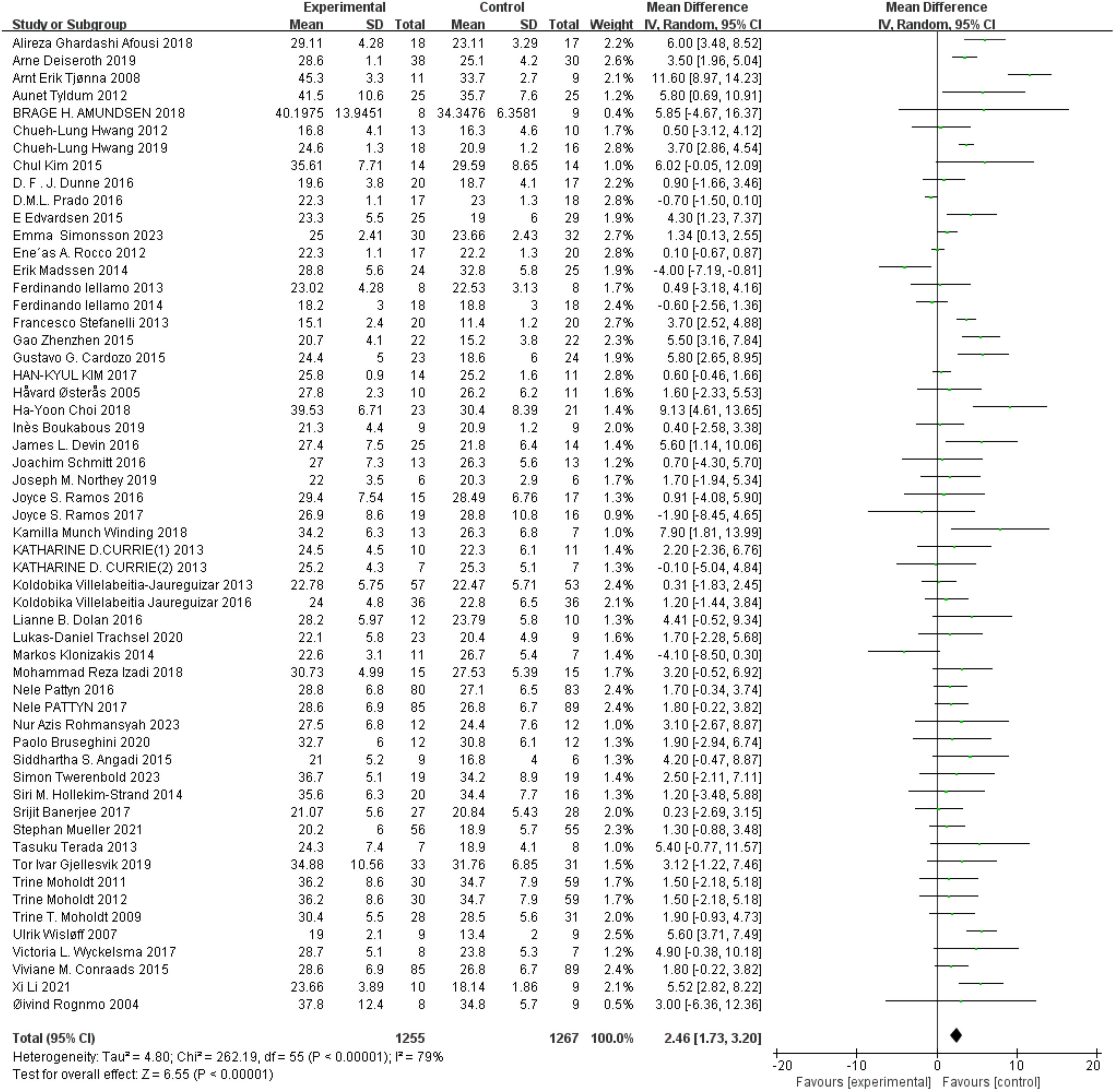
Forest plot of VO_2max_.

VO2_max_: A total of 56 (43, 44, 46, 49–51, 53, 54, 56–58, 60–63, 67, 68, 73–76, 78, 79, 83–85, 87, 91, 93–96, 98–104, 106–111, 113, 116, 117, 119–124, 126, 127) studies were included. Compared with the control group, HIIT had a significant effect on VO_2max_ in the older adult (MD: 2.46 mL min^−1^ kg^−1^, *p* < 0.00001), but there was statistical heterogeneity (*I* ^2^ = 79%) (**Figure 6**). Sensitivity analysis results suggested that the results of this study were robust. Funnel plots were basically symmetric, and there was no evidence of publication bias. Subgroup analysis showed that HIIT had a significant improvement in both the healthy and the sick older adult (*p* < 0.00001), but the improvement was greater in the sick older adult. There was no significant difference between subgroups (*p* > 0.05).

**Figure 7:**
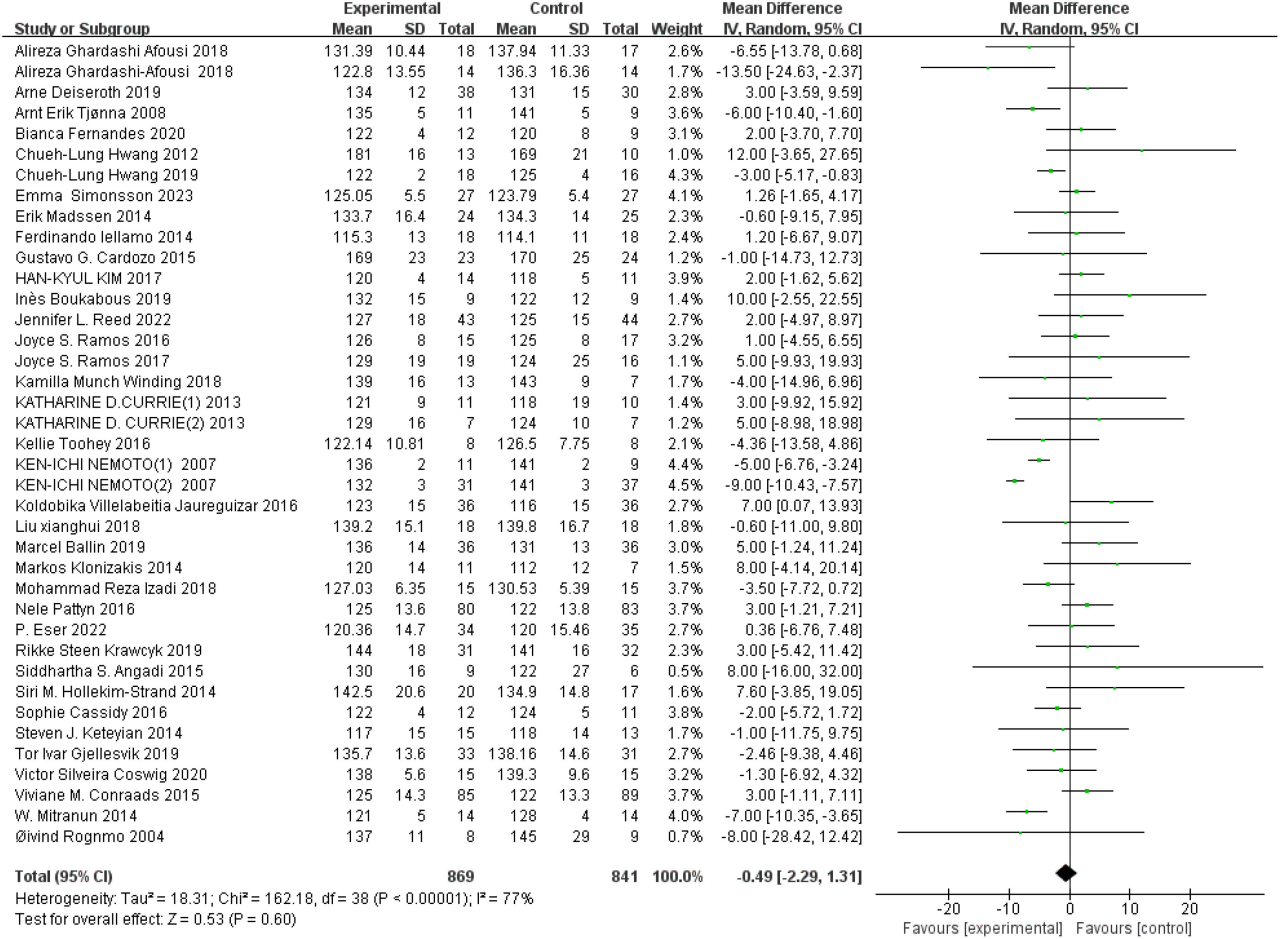
Forest plot of SBP.

SBP: A total of 38 (44, 45, 49, 51, 52, 54–58, 64, 65, 68, 69, 73–75, 78, 82, 84, 85, 91, 92, 97, 101, 103–105, 107, 110–112, 117–119, 124, 126, 128) studies were included. Compared with the control group, HIIT had no significant effect on SBP in the older adult (MD: −0.49 mmHg, *p* = 0.60) (**Figure 7**). Sensitivity analysis suggested that the results were robust. Funnel plots were basically symmetric, and there was no evidence of publication bias.

**Figure 8:**
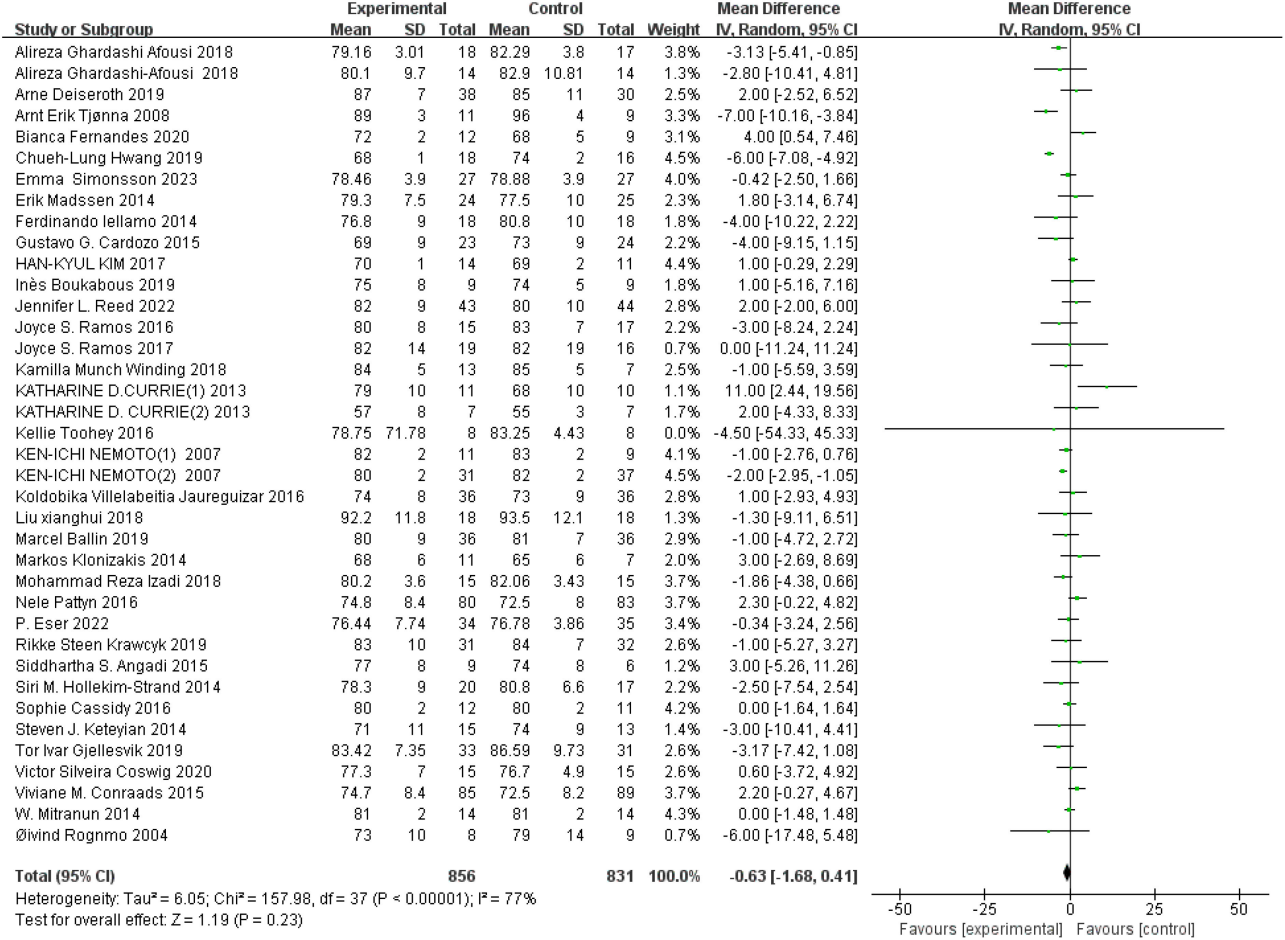
Forest plot of DBP.

DBP: A total of 37 (44, 45, 49, 51, 52, 54–58, 64, 65, 68, 69, 73, 75, 78, 82, 84, 85, 91, 92, 97, 101, 103–105, 107, 110–112, 117–119, 124, 126, 128) studies were included. Compared with the control group, HIIT had no significant effect on DBP in the older adult (MD: −0.63 mmHg, *p* = 0.23) (**Figure 8**). Sensitivity analysis suggested that the results were robust. Funnel plots were basically symmetric, and there was no evidence of publication bias.

**Figure 9:**
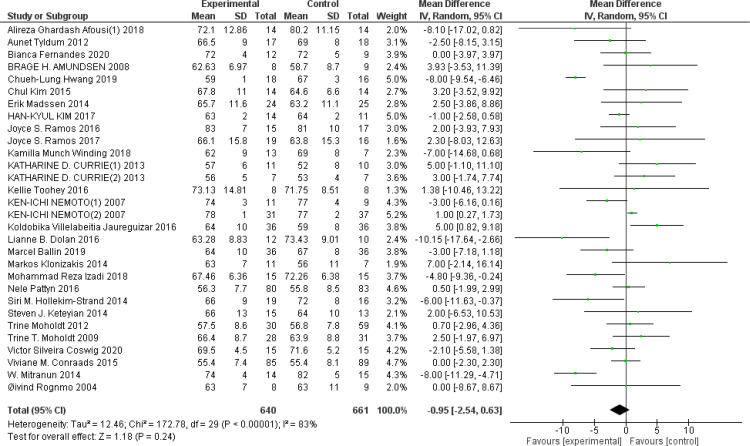
Forest plot of HR_rest_.

HR_rest_: A total of 29 (43, 45, 54–57, 61, 65, 68, 73, 78, 79, 82–85, 91–93, 95, 97, 101, 103, 104, 107, 111, 118, 121, 126) studies were included. Compared with the control group, HIIT had no significant effect on HR_rest_ in the older adult (MD: −0.95 BPM^−1^, *p* = 0.24) (**Figure 9**). Sensitivity analyses suggested that the results were robust. Funnel plots were basically symmetric, and there was no evidence of publication bias.

**Figure 10:**
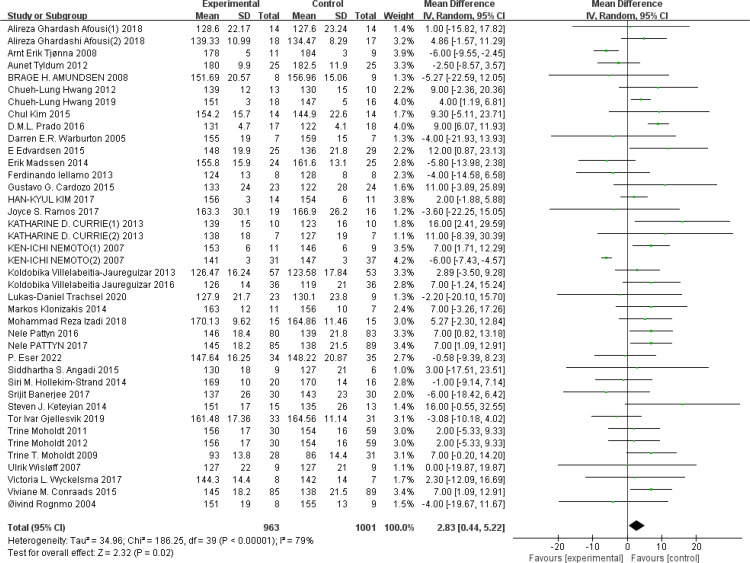
Forest plot of HR_max_.

HR_max_: A total of 39 (43, 44, 46, 51, 54, 56, 57, 63, 64, 68, 69, 73, 74, 76, 78, 79, 82–85, 91, 93–95, 97, 100–103, 107, 111, 117, 119–121, 123–125, 127) studies were included. Compared with the control group, HIIT significantly improved HR_max_ in the older adult (MD: 2.84 BPM^−1^, *p* = 0.02), but there was statistical heterogeneity (*I* ^2^ = 79%) (**Figure 10**) (see **Table 1**). Sensitivity analyses suggested that the results were robust. Funnel plots were basically symmetric, and there was no evidence of publication bias. Differences between subgroups suggest that disease type may be a source of heterogeneity (*p* < 0.0001).

**Figure 11:**
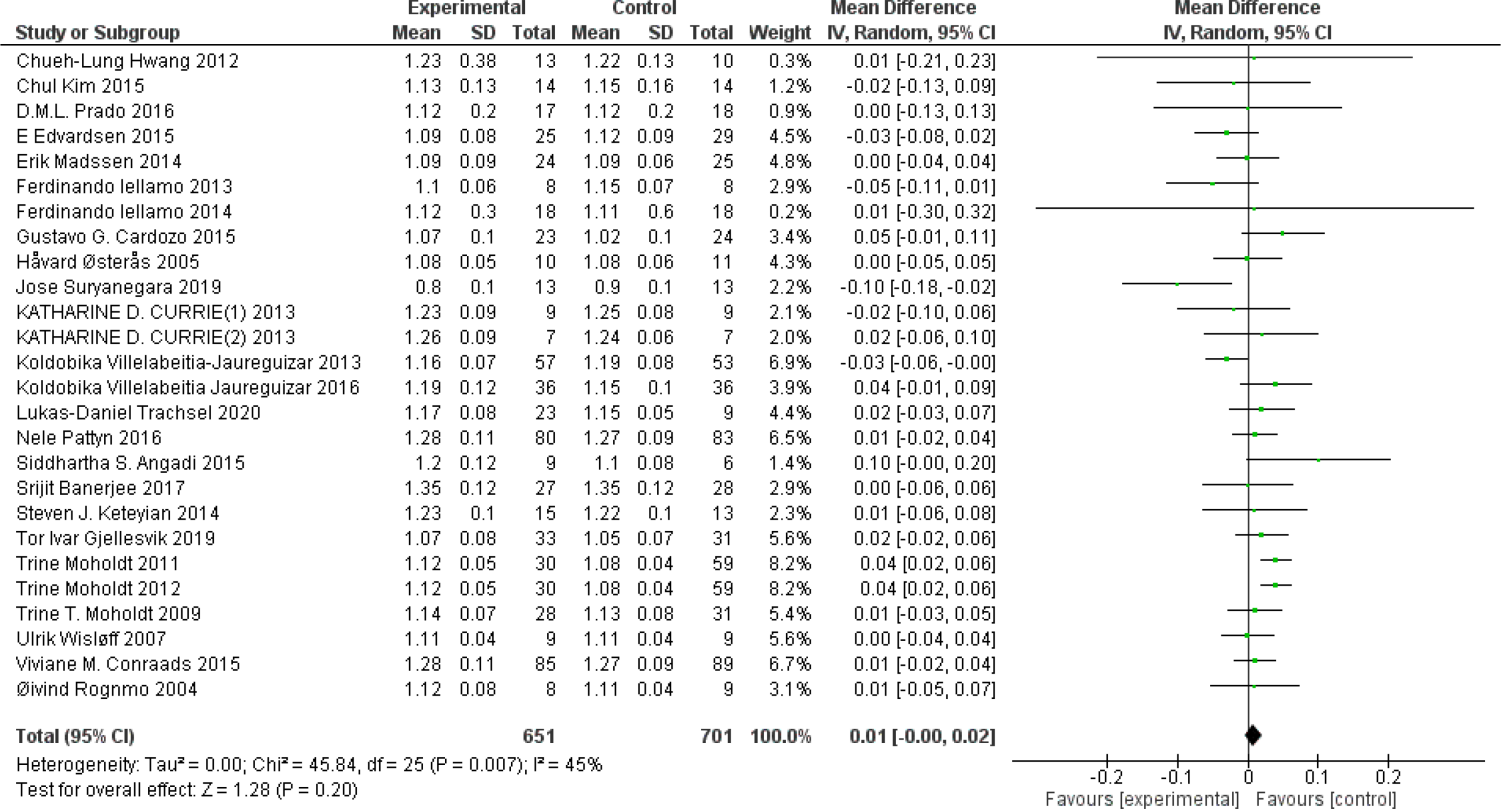
Forest plot of RER.

RER: A total of 26 (44, 46, 51, 54, 56, 57, 63, 74–76, 79, 82, 83, 91, 93–95, 99, 101, 102, 107, 114, 119, 120, 124, 127) studies were included. Compared with the control group, HIIT had no significant effect on RER in the older adult (MD: 0.01, *p* = 0.20) (**Figure 11**). After excluding studies by Villelabeitia-Jaureguizar et al. (124). *I* ^2^ was reduced to 33% and RER was significantly improved (MD: 0.01, *p* = 0.04). Sensitivity analysis results suggested that the results of this study lacked robustness. Funnel plots were basically symmetric, and there was no evidence of publication bias.

#### 3.3.3 Metabolic index

**Figure 12:**
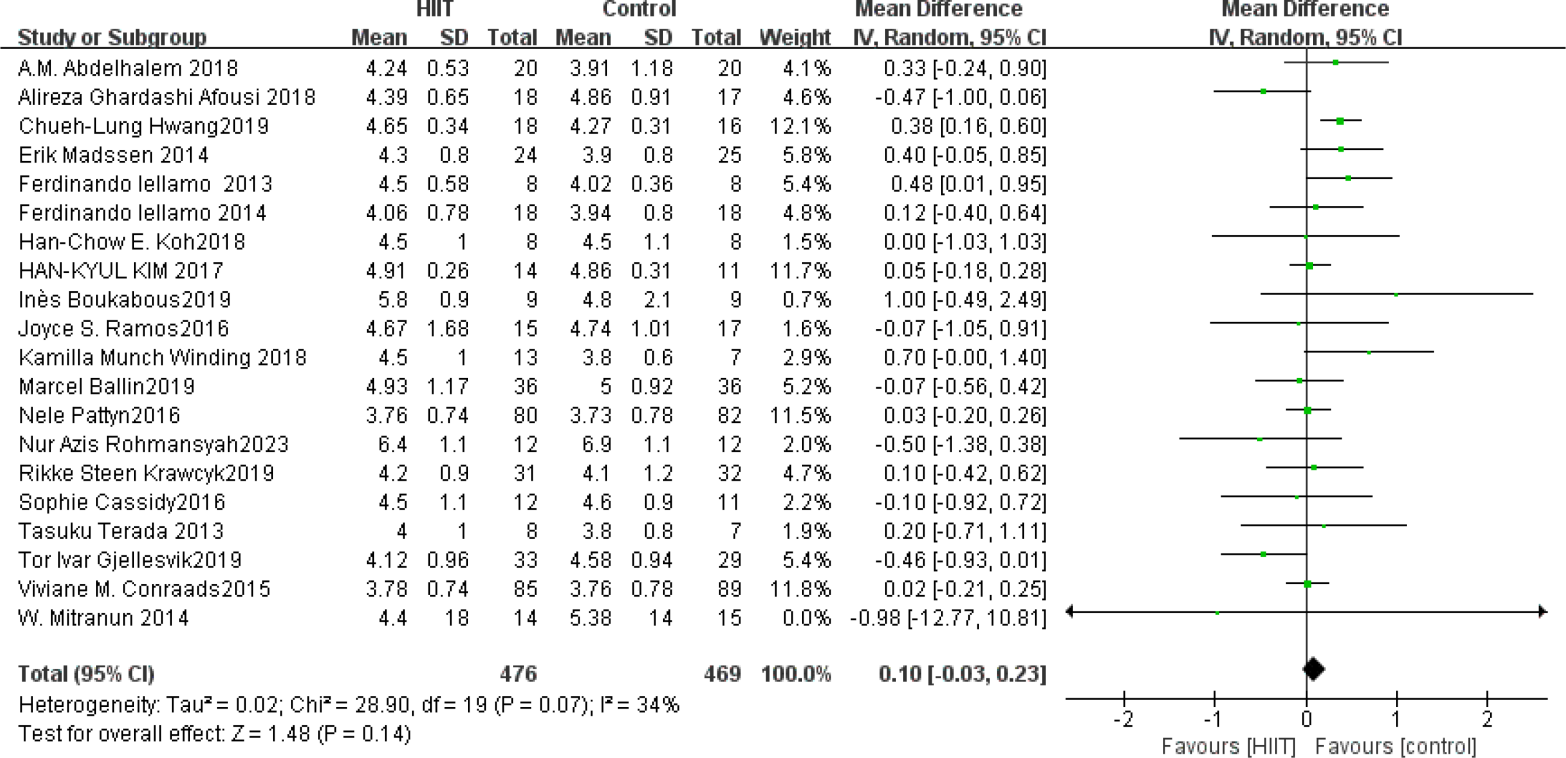
Forest plot of TC.

TC: A total of 20 (42, 45, 49, 52, 54, 68, 73, 75, 76, 84, 86, 91, 92, 101, 104, 108, 112, 116, 119, 126) studies were included. Compared with the control group, HIIT had no significant effect on TC in the older adult (MD: 0.10 mmol L^−1^, *p* = 0.14), but there was statistical heterogeneity (*I* ^2^ = 34%) (**Figure 12**). After excluding the study of Gjellesvik et al. (119), the heterogeneity decreased to 22% and significantly improved TC (MD: 0.13 mmol L^−1^, *p* = 0.006). Sensitivity analysis showed that the results of this study were not robust. Funnel plots were basically symmetric, and there was no evidence of publication bias.

**Figure 13:**
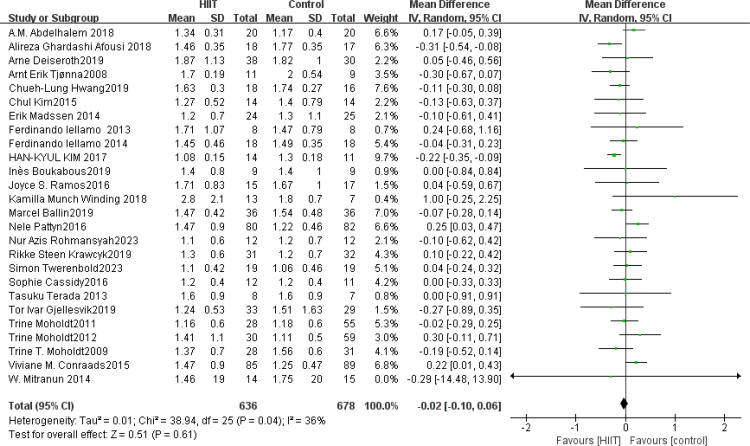
Forest plot of TG.

TG: A total of 26 (42, 45, 49, 52, 54, 58, 68, 73, 75, 76, 83, 84, 91–95, 101, 104, 108, 112, 116, 117, 119, 122, 126) studies were included. Compared with the control group, HIIT had no significant effect on TG (MD: −0.02 mmol L^−1^, *p* = 0.61) (**Figure 13**). Sensitivity analyses suggested that the results were robust. Funnel plots were basically symmetric, and there was no evidence of publication bias.

**Figure 14:**
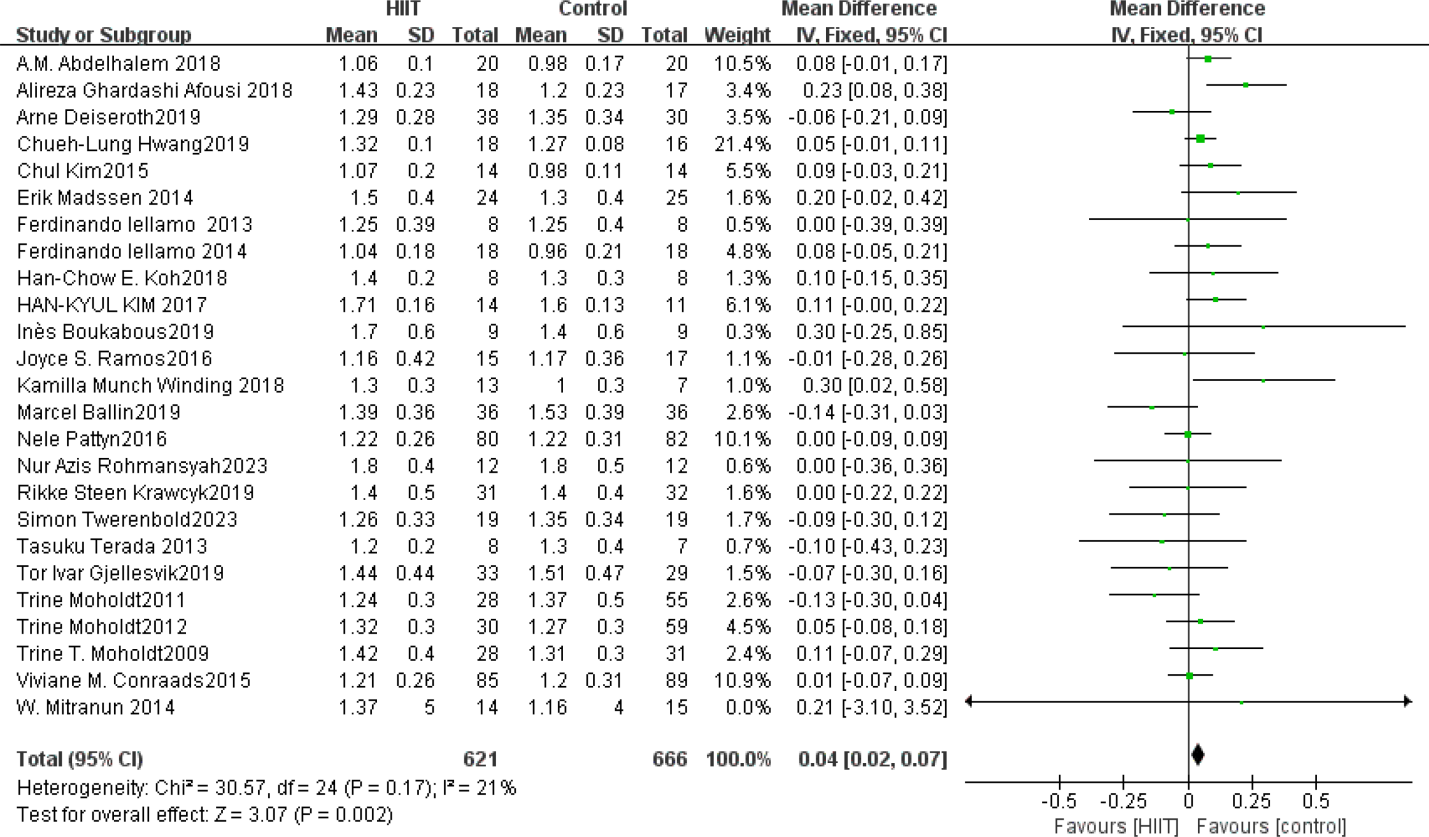
Forest plot of HDL.

HDL: A total of 25 (42, 45, 49, 54, 58, 68, 73, 75, 76, 83, 84, 86, 91–95, 101, 104, 108, 112, 116, 119, 122, 126) studies were included. Compared with the control group, HIIT significantly improved HDL (MD: 0.04 mmol L^−1^, *p* = 0.002) in the older adult, with low statistical heterogeneity (*I* ^2^ = 21%) (**Figure 14**). Sensitivity analysis showed that the results of this study were robust. Funnel plots were basically symmetric, and there was no evidence of publication bias. There was no significant difference between subgroups (*p* > 0.05).

**Figure 15:**
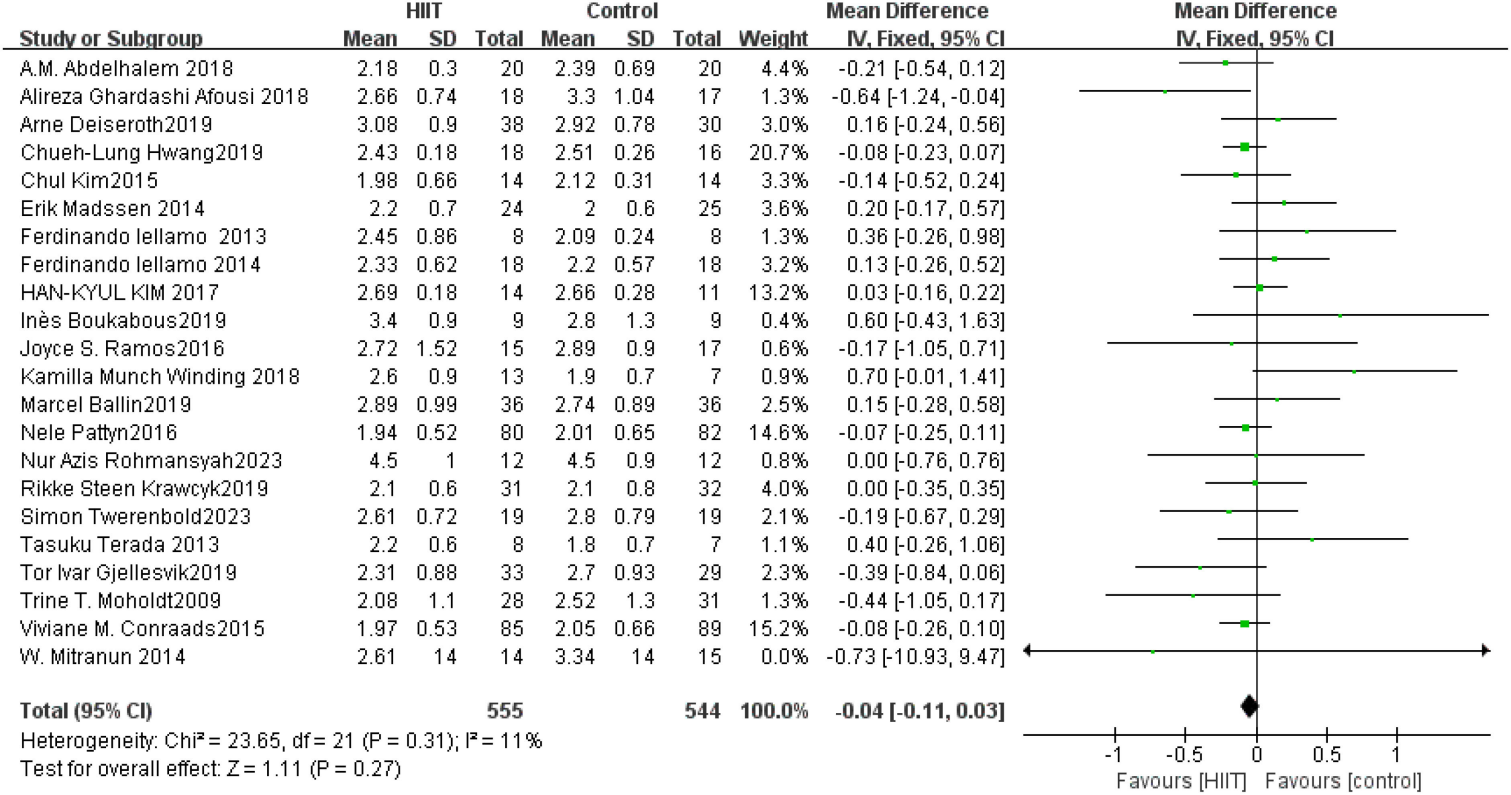
Forest plot of LDL.

LDL: A total of 22 (42, 45, 49, 54, 58, 68, 73, 75, 76, 83, 84, 91–93, 101, 104, 108, 112, 116, 119, 122, 126) studies were included. HIIT had no significant effect on LDL compared with the control group (MD: −0.04 mmol L^−1^, *p* = 0.27) (**Figure 15**). Sensitivity analyses suggested that the results were robust. Funnel plots were basically symmetric, and there was no evidence of publication bias.

**Figure 16:**
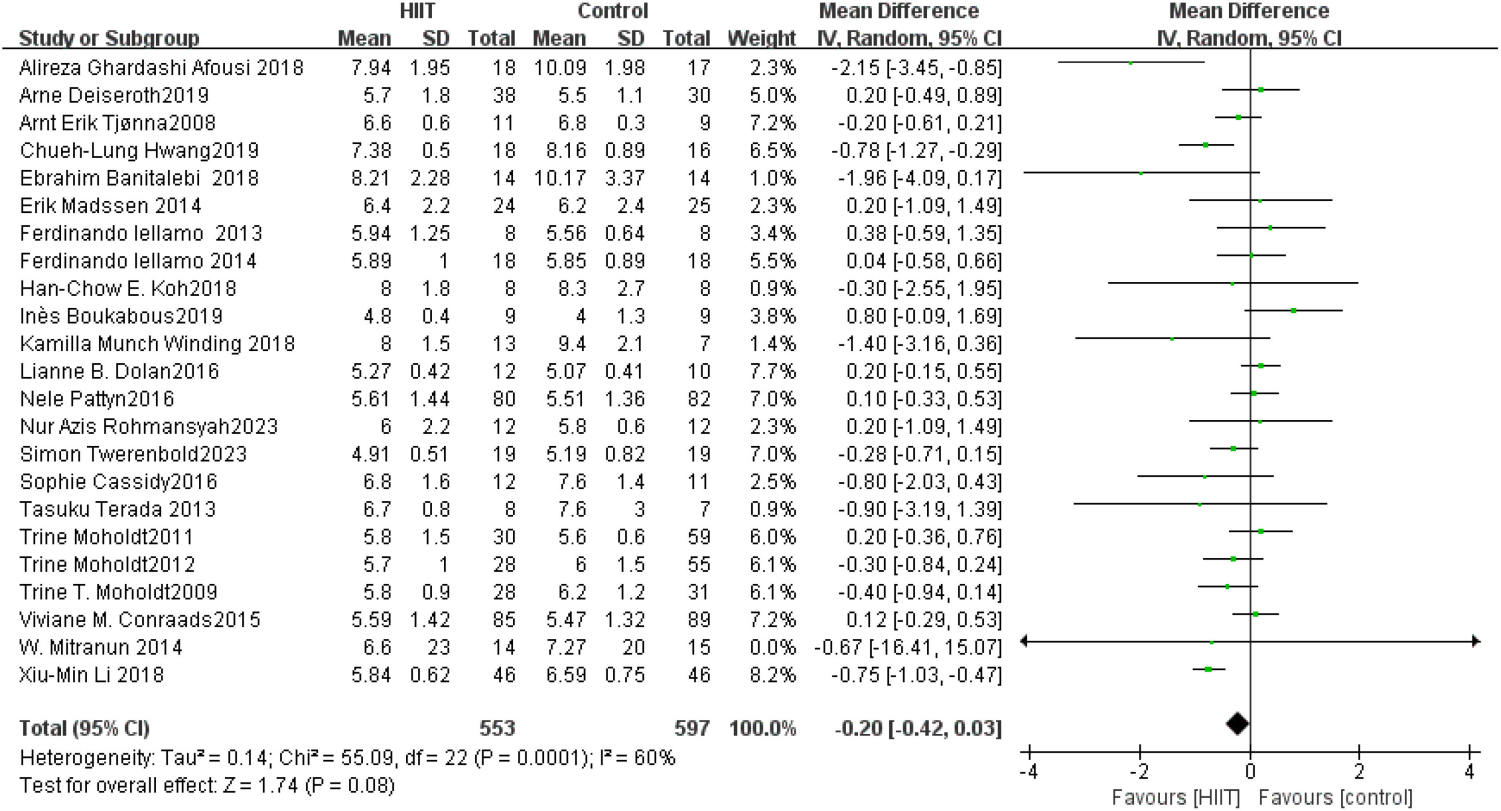
Forest plot of FPG.

FPG: A total of 23 (47, 49, 52, 54, 58, 61, 68, 73, 75, 76, 86, 88, 91–95, 101, 108, 116, 117, 122, 126) studies were included. Compared with the control group, HIIT had no significant effect on FPG in the older adult (MD: −0.20 mmol L^−1^, *p* = 0.08) (**Figure 16**). After excluding the study by Gjellesvik et al. (119), the heterogeneity decreased to 22% and significantly improved FPG (MD: −0.13 mmol L^−1^, *p* = 0.006). The results of sensitivity analysis suggested that the results of this study lacked robustness. Funnel plots were basically symmetric, and there was no evidence of publication bias.

## 4 Discussion

This meta-analysis examined the overall impact of HIIT on older adult health by analyzing 14 clinical indicators across three categories: body composition, cardiopulmonary function, and metabolism. To our knowledge, this study is the largest in the world to evaluate HIIT’s effects on older adult health, encompassing 87 eligible RCT studies involving 4,213 participants from various populations including healthy individuals as well as those with hypertension, CVD, diabetes, cancer, and other conditions; it would be pertinent to note that most of this population resides in the Northern Hemisphere. Our findings support the universal efficacy of HIIT in improving BF%, VO_2max_, HR_max_, and HDL levels among older adults; however, it does not affect BMI, SBP, DBP, HR_rest_, TG, and LDL levels. The results for WC, RER, TC, and FPG should be interpreted with caution due to their lack of robustness, which may be attributed to differences in disease type. These findings provide evidence for the use of HIIT in clinical or home health management as well as guidelines/recommendations for the older adult.

### 4.1 Body shape index

Owing to the intricate relationship between body shape and health as well as disease (129), it serves as a primary indicator for assessing the health status of older individuals. Two extensive cohort studies have demonstrated that both high BMI and BF% along with low BMI elevate the risk of mortality (130, 131). Therefore, it is crucial for older adult individuals to effectively manage their BMI and reasonably reduce their BF%. This meta-analysis aims to elucidate the impact of HIIT on three clinical parameters related to body shape in older adults. The results of the meta-analysis demonstrated that HIIT effectively improved BF% in older adults, which is consistent with the findings reported by Donghai et al. (132) and McLeod et al. (33), but contradicts the conclusions drawn from other studies (26, 29–32, 34–37). Unlike previous studies that primarily included individuals with normal weight as baseline data, the older adults we enrolled were mostly overweight or obese (BMI ≥25), although we did not restrict inclusion and exclusion criteria. HIIT stimulates lipolysis by increasing catecholamines and growth hormone (133). Meanwhile, HIIT improved BF%, including body composition and lipid profile, by increasing HDL in human and animal models (134–137), a mechanism consistent with our meta results for biochemical metrics. The meta-analysis results indicated insufficient evidence regarding the impact of HIIT on WC and BMI in older adults, aligning with most previous systematic reviews (26, 29–32, 34–37). To further assess the stability of these combined results, a sensitivity analysis was performed. The pooled results for BMI were stable, and for WC after excluding the study conducted by Tjønna et al. (117), *I* ^2^ decreased from 80% to 48%, This change may be related to two aspects. First, the difference in the included population: Tjønna et al. (117) included the older adult with metabolic syndrome, while other studies included healthy people or people with one disease. Second, unlike BMI, WC directly reflects the characteristics of fat distribution. Given the relationship between the pathological mechanism of metabolic syndrome and visceral fat function, the effect size may be nonlinear with other populations (138). These factors may exacerbate heterogeneity. It is worth noting that, excluding the study by Madssen et al. (91), there was no significant change in *I* ^2^; however, WC exhibited a reversal (*p* = 0.04). Further analysis revealed that the exercise supervision implementation rate in the study was only 1/3, and this low compliance resulted in an increase in WC after HIIT intervention. This finding may further elucidate the insufficient effect of HIIT on WC combined results. Therefore, caution should be exercised when interpreting whether HIIT can improve WC in the older adult due to the lack of robustness of these results. HIIT did not significantly improve BMI in the older adult. The results of the study by Donghai et al. (132) suggested that although HIIT decreased BF% in the older adult, the increase in lean body mass did not change their total body weight significantly, resulting in no significant difference in BMI before and after exercise. This lack of significant difference in measurement results due to adaptive changes in the body is not related to the study hypothesis.

### 4.2 Cardiopulmonary function index

The pleiotropic effects of maintaining a high level of cardiopulmonary function on the health of the older adult have been widely recognized (139), and it has been identified as a priority for promoting the health of the older adult by the World Health Organization (WHO) (12). A total of six cardiopulmonary function indicators were included in this meta-analysis to evaluate the effects of HIIT on cardiopulmonary function in the older adult. The existing evidence shows that HIIT can effectively improve VO_2max_ and HR_max_ in the older adult. The analysis of 55 studies showed that the increase of VO_2max_ by 2.46 mL/kg/min is very significant, and VO_2max_ is recognized as the gold standard of cardiopulmonary function. A 44-year follow-up study showed that VO_2max_ was inversely associated with the risk of cancer, cardiovascular events, and all-cause mortality (140), and each 1-MET increase reduced cardiovascular events by 15% and all-cause mortality by 13% (141). Subgroup analysis found that the effect of HIIT on VO_2max_ in the older adult was generalized and was independent of the intervention period and the health of the subjects. In addition, HR_max_ is also one of the important indexes to evaluate the cardiopulmonary function of the older adult, and it is positively correlated with VO_2max_. The improvement of HR_max_ is consistent with VO_2max_, but the improvement of HR_max_ is less extensive than VO_2max_. Although the aim of this study was not to investigate the underlying physiological mechanisms of HIIT, based on the current evidence, we highlighted some mechanisms related to the obtained results, such as high physiological stress caused by HIIT, high recruitment pattern of type I and type II fibers, and intense muscle contraction during exercise, leading to imbalance of the ATP/ADP relationship, increased PGC1-α activation and, thus, increased PGC1-α activation, and strong body VO_2max_ (134, 142, 143). The results of this study show that there is insufficient evidence for HIIT to improve blood pressure in the older adult, which is consistent with the results of previous studies (30–37, 132), but in sharp contrast to the study by Du et al. (29), the difference in conclusions may be related to the training method [isometric exercise has a better effect on blood pressure change than other exercise methods (144)] and the sensitivity of the subjects to HIIT. Therefore, the effects of aging on HIIT-induced fitness may be interesting to investigate in the future. Blood pressure is the most important modifiable risk factor for all-cause morbidity and mortality; however, our evidence found that HIIT had no significant effect on SBP and DBP, and neither changed significantly after individual removal, possibly because the majority of non-hypertensive people we included undermined this effect. After the study by Villelabeitia-Jaureguizar et al. (124) was excluded by RER, the results were reversed (*p* = 0.04). Since this study compared the improvement difference between moderate-intensity exercise and HIIT, the effect of HIIT was reduced. The results for RER need to be interpreted with caution, given the lack of robustness of the sensitivity analyses. Unfortunately, there is insufficient evidence on the effect of HIIT on HR_rest_ in the older adult; thus, it was omitted from this meta-analysis.

### 4.3 Metabolic index

Metabolic abnormalities can cause a variety of chronic diseases, including obesity, CVD, diabetes, and cancer, which bring huge public health problems and medical burdens (145). Metabolic disorders are often more serious in the older adult. Priority should be given to reducing the related risks in the older adult at this stage while preventing them. A total of five metabolic indicators were included in this meta-analysis, and the existing evidence showed that HIIT effectively improved HDL, which was in sharp contrast with previous studies (26, 29, 31) probably because previous studies were based on comparing the differences between HIIT and moderate-intensity exercise, while most of our work was based on the comparison between HIIT and blank control. Because of the strong association between dyslipidemia and CVD, HIIT is of great significance for the improvement of HDL levels. However, HIIT had no significant effect on TG and LDL, which is consistent with previous studies (33, 37). The results of TC and FPG were reversed after excluding the study of Gjellesvik et al. (119), which may be due to the differences in the baseline levels of our included studies, two types of exercise in some studies, and the use of drugs to treat metabolic abnormalities or affect metabolic indicators. These factors may reduce the improvement effect of HIIT on metabolic indicators. It also indicates the lack of robustness of the TC and FPG results, and this result needs to be interpreted with caution.

### 4.4 Adverse events and compliance

Among the included studies, 25 studies provided comprehensive descriptions of medical supervision, while 29 studies lacked detailed information in this regard, and 33 studies did not mention it at all. Additionally, withdrawal from the intervention was reported in 24 studies due to reasons such as familial obligations, personal preferences, and other factors, involving a total of 196 participants, accounting for approximately 4.65% of the overall sample size. Adverse events were documented in 46 cases with an incidence rate of approximately 1.09%. Importantly, none of these adverse events were attributed to HIIT.

### 4.5 Limitations

The large sample size and high heterogeneity observed in our study were expected due to differences in methodology and study subjects, as the range of studies included all older adults except those with contraindications to exercise. Therefore, subgroup analyses and sensitivity points were conducted to assess the role and stability of the pooled results. Sensitivity analysis revealed that WC, RER, TC, and FPG lacked robustness. Despite implementing a rigorous search strategy, language bias was inevitable as only Chinese and English literature was retrieved within our constraints. Furthermore, variations in exercise equipment, interval time, and intervention duration among the HIIT studies included prevented us from conducting subgroup analysis; thus, specific exercise doses cannot be recommended at this stage. Lastly, our included studies did not assess medication use or baseline/daily physical activity, which are critical factors for evaluating the hypothesis that HIIT improves health in older adults. Additionally, we lacked data on other influencing factors such as gender and exercise capacity. Finally, it should be emphasized that most of the included samples were from the Northern Hemisphere, and the unbalanced sample distribution may limit the promotion of HIIT in the Southern Hemisphere.

## 5 Conclusion

Current evidence suggests that HIIT is associated with improvements in BF%, VO_2max_, HR_max_, and HDL, but not in BMI, SBP, DBP, HR_rest_, TG, and LDL in the older adult. In addition, owing to the differences in subjects, baseline, exercise dose, and sample distribution, the results of WC, RER, TC, and FPG lack robustness, and the conclusions need to be further studied and discussed. These findings provide data support for a comprehensive interpretation of the role of HIIT in promoting physical health in the older adult. In view of the significant clinical benefits of HIIT in improving the VO_2max_ of the older adult, HIIT can be used as an effective means to improve the cardiopulmonary function of the older adult under the premise of medical supervision. However, the optimal exercise dose of HIIT is still uncertain, which requires future multi-center, large-scale, and high-quality studies as well as long-term prospective investigations to verify these results, and also needs to consider the global distribution of samples.

## Data Availability

The original contributions presented in the study are included in the article/**Supplementary Material**. Further inquiries can be directed to the corresponding author.
