# Supplementary material for: Effects of high-intensity interval training on physical morphology, cardiopulmonary function, and metabolic indicators in older adults: a systematic review and meta-analysis
Source: Front Endocrinol (Lausanne). 2025 Mar 25;16:1526991. doi: 10.3389/fendo.2025.1526991 (PMC11975580; doi:10.3389/fendo.2025.1526991)
Supplement: Supplementary file 1 [file Table1.docx]

**Additional file 1** Search Strategy.

**Pubmed:**

| **Items** | **Search Terms** | **Search Results** |
| --- | --- | --- |
| #1 | ((aged[MeSH Terms]) OR (elderly[Title/Abstract])) OR (older[Title/Abstract]) | 3779678 |
| #2 | ((High-Intensity Interval Training[MeSH Terms]) OR (HIIT[Title/Abstract])) OR (High-Intensity Intermittent Exercise[Title/Abstract]) | 3336 |
| #3 | (((randomized controlled trial[MeSH Terms]) ) OR (RCT[Title/Abstract])) OR (randomized clinical[Title/Abstract]) | 245704 |
| #4 | #1 AND #2 AND #3 | 40 |

**Cochrane Library:**

| **Items** | **Search Terms** | **Search Results** |
| --- | --- | --- |
| #1 | (aged):ti,ab,kw OR (elderly):ti,ab,kw OR (older):ti,ab,kw | 648333 |
| #2 | (High-Intensity Interval Training):ti,ab,kw OR (HIIT):ti,ab,kw OR (High-Intensity Intermittent Exercise):ti,ab,kw | 3856 |
| #3 | (randomized controlled trial):ti,ab,kw OR (RCT):ti,ab,kw OR (randomized clinical):ti,ab,kw | 852758 |
| #4 | #1 and #2 and #3 | 801 |

**Embase**:

| **Items** | **Search Terms** | **Search Results** |
| --- | --- | --- |
| #1 | 'aged'/exp OR 'aged' OR 'elderly'/exp OR 'elderly' OR 'older' | 6040346 |
| #2 | 'high-intensity interval training' OR 'hiit' OR 'high-intensity intermittent exercise' | 5925 |
| #3 | **'**randomized controlled trial' OR 'rct' OR 'randomized clinical' | 1088023 |
| #4 | #1 and #2 and #3 | 694 |

**Web of science:**

| **Items** | **Search Terms** | **Search Results** |
| --- | --- | --- |
| #1(participents) | TS=(adolescent OR Adolescence OR Teens OR Teenagers OR Youths OR Female Adolescents OR Male Adolescents) | 2837751 |
| #2(intervention) | TS=(High-Intensity Interval Training OR High-Intensity Interval OR High-Intensity Intermittent OR High-Intensity Intermittent Exercises OR Sprint Interval Trainings OR hiit) | 5171 |
| #3(study) | TS=(randomized controlled trial OR RCT OR Randomized OR Randomized Clinical OR Controlled Clinical Trials) | 1435301 |
| #4 | #1 AND #2 AND #3 | 345 |

**SCOPUS :**

| **Search Terms** | **Search Results** |
| --- | --- |
| TITLE-ABS-KEY ( "Aged" OR "Elderly" OR "Older" ) AND TITLE-ABS-KEY ( "High-Intensity Interval Training" OR "HIIT" OR "High-Intensity Intermittent Exercise" ) AND TITLE-ABS-KEY ( "randomized controlled trial" OR "RCT" OR "randomized clinical" ) | 738 |

**CNKI：**

| **Search Terms** | **Search Results** |
| --- | --- |
| (SU="老年人" OR SU="高龄老人" OR SU="老年群体") AND (SU="高强度间歇训练" OR SU="间歇训练" OR SU="有氧间歇训练" OR SU="高强度有氧间歇训练") AND (SU="随机对照试验" OR SU="随机化" OR SU="随机分配" OR SU="对照试验" OR SU="对照") | 3 |

**Wanfang：**

| **Search Terms** | **Search Results** |
| --- | --- |
| （（“老年人” and “高强度间歇训练”and “随机” ）or （“老年群体”and “间歇训练”and “随机对照”）or （“老人”and “高强度间歇训练”and “随机”）） | 2 |
