# Supplementary material for: Effects of high-intensity interval training on physical morphology, cardiopulmonary function, and metabolic indicators in older adults: a systematic review and meta-analysis
Source: Front Endocrinol (Lausanne). 2025 Mar 25;16:1526991. doi: 10.3389/fendo.2025.1526991 (PMC11975580; doi:10.3389/fendo.2025.1526991)
Supplement: Supplementary file 2 [file Table2.docx]

**Additional file 2** Table 1 Basic features of included studies

| **Author** | **Year** | **Country** | **Lesion** | **Age** | | **Sample** | | **Intervention measure** | | **Intervention** | | **Outcomes** |
| --- | --- | --- | --- | --- | --- | --- | --- | --- | --- | --- | --- | --- |
|  |  |  |  | **experimental group** | **control group** | **experimental group（male/female）** | **Control group（male/female）** | **experimental group** | **control group** | **experimental group** | **control group** |  |
| Arnt et al.[34] | 2008 | Norway | Metabolic syndrome | 55.3±13.2 | 49.6±9.0 | 11(4/7) | 9(5/4) | mode of motion: running  exercise time: 25 mins  exercise frequency: three times per week  exercise intensity: 90%VO_2_max | mode of motion: daily activities | 16 weeks | 16 weeks | 1,3,4,5,6,8,10,14 |
| Alireza et al.[35] | 2018 | Iran | Coronary artery disease | 53.90±3.44 | 58.80±4.41 | 14(14/0) | 14(14/0) | mode of motion: running  exercise time: 20 mins  exercise frequency: three times per week  exercise intensity: 85-95%HRmax | mode of motion: daily activities | 6 weeks | 6 weeks | 5,6,7,8 |
| Alireza et al.[36] | 2018 | Iran | Type 2 diabetes | 54.78 ± 6.19 | 54.24 ± 5.61 | 18(9/9) | 17(9/8) | mode of motion: cycling  exercise time: 42 mins  exercise frequency: three times per week  exercise intensity: 85-90% HRmax | mode of motion: daily activities | 12 weeks | 12 weeks | 1,5,6,10,11,12,13,14 |
| A.M. et al.[37] | 2018 | Egypt | Left ventricular dysfunction | 54.65 ± 7.63 | 51.95 ± 8.07 | 20 | 20 | mode of motion: intensive exercise  exercise time: 30-35 mins  exercise frequency: two times per week  exercise intensity: 85-95% HRmax | mode of motion: running  exercise time: 30-35mins  exercise frequency: two times per week  exercise intensity: 40-60%HRmax | 12 weeks | 12 weeks | 10,11,12,13 |
| Arne et al.[38] | 2019 | United States | Healthy | 58 ± 6 | 59 ± 7 | 38 | 30 | mode of motion: walking  exercise time: 25 mins  exercise frequency: three times per week  exercise intensity: 80-90%HRmax | mode of motion: healthy and sedentary | 12 weeks | 12 weeks | 1,3,4,5,6,7,11,12,13,14 |
| Brage et al.[39]. | 2008 | Norway | Coronary heart disease | 63±11 | 61±7 | 8(6/2) | 9(8/1) | mode of motion: walk  exercise time: 25 mins  exercise intensity: 80-90% VO_2_max | mode of motion: walk  exercise time: 41 mins  exercise intensity: 50-60%VO_2_max | 10 weeks | 10 weeks | 4,5,6,7,8 |
| Bianca et al.[40] | 2020 | Canada | Parkinson | 67.6 ± 8.9 | 69.8 ± 7.7 | 12 (7/5) | 9 (6/3) | mode of motion: running  exercise time: 25 mins  exercise frequency: three times per week | mode of motion: moderate intensity continuous training | 12 weeks | 12 weeks | 5,6,7 |
| Céline et al.[41] | 2012 | France | Heart failure | 54±9 | 55±12 | 12(6/6) | 14(7/7) | mode of motion: interval training  exercise time: 42 mins  exercise frequency: five days per week  exercise intensity: 80%-95% VO_2_max | mode of motion: continuous training  exercise time: 50 mins  exercise frequency: five days per week | 8 weeks | 8 weeks | 5,6 |
| Chueh-Lung et al.[42] | 2012 | China | Cancer | 61.0±6.3 | 58.5±8.2 | 11(7/4) | 13(5/8) | mode of motion: running and cycling.  exercise time: 15-25 mins  exercise frequency: three sessions per week exercise intensity: 80% VO_2_max | mode of motion: daily activities | 8 weeks | 8 weeks | 1,4,5,7,9 |
| Chul et al.[43] | 2015 | Korea | Myocardial infarct | 57±11.58 | 60.2±13.64 | 14 (12/2) | 14（10/4） | mode of motion: walking on a treadmill  exercise time: 45 mins  exercise frequency: three times per week  exercise intensity: 85%-95%HRR | mode of motion: walking on a treadmill  exercise time: 60 mins  exercise frequency: three times per week  exercise intensity: 70%-85%HRR | 6weeks | 6weeks | 4,7,8,9,10,11,12,13 |
| Chetna et al.[44] | 2019 | Switzerland | Cancer | 64 ±13 | 64 ±10 | 55(41/14) | 55(50/15) | mode of motion: cycling  exercise time: 10 mins  exercise frequency: three times per week  exercise intensity: 50%-100%HRmax | mode of motion: usual care | 4 weeks | 4 weeks | 4 |
| Christopher et al.[45] | 2019 | UK | Healthy | 61.9 (50–81) | 62.8 (50–74) | 18(11/7) | 18(10/8) | mode of motion: intensive exercise  exercise time: 12-20 mins  exercise frequency: two times per week  exercise intensity: 90% HRmax | mode of motion: daily activities | 12 weeks | 12 weeks | 4 |
| Chueh-Lung et al.[46] | 2019 | United States | Type 2 diabetes | 65 ± 2 | 61 ± 2 | 23 | 16 | mode of motion: running  exercise time: 25 mins  exercise frequency: four times per week  exercise intensity: 90%HRmax | mode of motion: daily activities | 8 weeks | 8 weeks | 1,2,3,4,5,6,7,8,10,11,12,13 |
| Darren et al.[47] | 2005 | Canada | Coronary heart disease | 55 ± 7 | 57±8 | 7 | 7 | mode of motion: running  exercise time: 30 mins exercise frequency: two times per week  exercise intensity: 90% HRmax(85% - 95%VO_2_max) | mode of motion: running  exercise time: 30 mins exercise frequency: two times per week  exercise intensity: 65%HRmax | 16 weeks | 16 weeks | 8 |
| D. F . J. et al.[48] | 2016 | UK | Hepatectomy | 61 (56–66) | 62 (53–72) | 20 | 17 | mode of motion: cycle ergometer  exercise time: 30 mins  exercise frequency: three times per week  exercise intensity: ＞90% VO_2_max | mode of motion: daily activities | 4 weeks | 4 weeks | 4 |
| D.M.L. et al[49] | 2016 | Brasil | Coronary heart disease | 56.5 ± 2.7 | 61.3 ± 2.2 | 17 | 18 | mode of motion: running  exercise time: 42 mins  exercise frequency: three times per week  exercise intensity: seven sets of 3 min at RCP and seven sets of 3 min of exercise at VAT intensity | mode of motion: running  exercise time: 50 mins  exercise frequency: three times per week  exercise intensity: 50-min of treadmill exercise at VAT intensity | 12weeks | 12weeks | 1,4,8,9 |
| Ene´as et al.[50] | 2012 | Brazil | Coronary heart disease | 62.5±2.0 | 56.5±3.0 | 20(15/5) | 17(14/3) | mode of motion: moderate intensity exercise  exercise time: 21 mins  exercise frequency: three times per week  exercise intensity: 80-90% VO_2_max | mode of motion: running  exercise time: 50 mins  exercise frequency: three times per week  Exercise intensity: 70-80%VO_2_max | 12 weeks | 12 weeks | 1,4 |
| Erik et al.[51] | 2014 | Norway | Coronary artery disease | 64.4 (47–78) | 58.5 (42–71) | 24(18/6) | 25(18/7) | mode of motion: HIIT  exercise time: 25 mins  exercise frequency: three times per week  exercise intensity: 85-95% HRmax | mode of motion: daily activities | 48weeks | 48weeks | 1,3,4,5,6,7,8,9,11,12,13,14 |
| Edvardsen et al.[52] | 2015 | Norway | Cancer | 64.4±9.3 | 65.9±8.5 | 25 | 29 | mode of motion: walking on a treadmill  exercise time: 60 mins  exercise frequency: three times per week  exercise intensity: 80-95% VO_2_max | mode of motion: hip and knee horizontal stretching exercises  exercise time: 60 mins  exercise frequency: three times per week | 20weeks | 20weeks | 1,4,8,9 |
| Ebrahim et al.[53] | 2018 | Iran | Type 2 diabetes | 55.07±5.29 | 55.07±5.29 | 17(0/17) | 18(0/18) | mode of motion: cycling  exercise time: 13 mins | mode of motion: daily activities | 10 weeks | 10 weeks | 1,2,14 |
| Emma et al.[54] | 2023 | Sweden | Healthy | 69.7±3.15 | 69.6±2.8 | 34 | 34 | mode of motion: indoor bicycles  exercise time: 20 mins  exercise frequency: two times per week  exercise intensity: 85 RPM | mode of motion: indoor bicycles  exercise time: 40 mins  exercise frequency: two times per week  exercise intensity: 85 RPM | 12 weeks | 12 weeks | 4,5,6 |
| Ferdinando et al.[55] | 2013 | Italy | Heart failure | 62.2± 8 | 62.6±9 | 8(8/0) | 8(8/0) | mode of motion: walk  exercise time: 30-45 mins  exercise frequency: two-five times per week  exercise intensity: 75–80%HRR | mode of motion: walk  exercise time: 30-45 mins  exercise frequency: two-five times per week  exercise intensity: 45-60%HRR | 12 weeks | 12 weeks | 4,8,9,10,11,12,13,14 |
| Francesco et al.[56] | 2013 | Italy | Cancer | 65.5±7. | 64.8±7.3 | 20 | 20 | mode of motion: running and cycling  exercise time: three hours  exercise frequency: five days per week | mode of motion: daily activities | 3 weeks | 3 weeks | 4 |
| Ferdinando et al.[57] | 2014 | Italy | Heart failure | 67.2 ± 6 | 68.4 ± 8 | 18(16/2) | 18(15/3) | mode of motion: HIT  exercise time: 25 mins  exercise frequency: three times per week  exercise intensity: 80-85% of HRR | mode of motion: MIT  exercise time: 30-45mins  exercise frequency: three times per week  exercise intensity: 45-60% of HRR | 12weeks | 12weeks | 4,5,6,10,  11,12,13,14 |
| Fang et al.[58] | 2020 | China | Healthy | 64.90±3.45 | 65.00±4.12 | 10(7/3) | 9(5/4) | mode of motion: running  exercise time: 16 mins  exercise frequency: three times per week  exercise intensity: 85-90% VO_2_max | mode of motion: running  exercise time: 16 mins  exercise frequency: three times per week  exercise intensity: 85-90% VO_2_max | 12 weeks | 12 weeks | 1,2 |
| Ferdinando et al.[59] | 2021 | Switzerland | Hypertension | 64.5 ± 7.2 | 66 ± 4.7 | 12 | 12 | mode of motion: running  exercise time: 15 mins  exercise intensity: 80-95%VO_2_max | mode of motion: running  exercise time: 45 mins  exercise intensity: 55-70% VO_2_max | 12 weeks | 12 weeks | 4, |
| Gao et al.[60] | 2015 | China | Coronary artery disease | 59.4±7.9 | 60.4±8.6 | 22(18/4) | 22(17/5) | mode of motion: treadmill training  exercise time: 40 mins  exercise frequency: three times per week  exercise intensity: 80%PP | mode of motion: daily activities | 12weeks | 12weeks | 4 |
| Gustavo et al.[61] | 2015 | Brazil | Coronary heart disease | 56 ± 12 | 64 ± 12 | 24 | 24 | mode of motion: aerobic training  exercise time: 40 mins  exercise frequency: three times per week  exercise intensity: 70-90%HRmax | mode of motion: daily activities | 16weeks | 16weeks | 4,5,6,8,9 |
| Håvard et al.[62] | 2005 | Norway | Healthy | 68.9 ± 2.8 | 70.5 ± 3.0 | 10(4/6) | 11(4/7) | mode of motion: running  exercise time: 60 mins exercise frequency: three times per week exercise intensity: 85% to 95% HRmax | mode of motion: daily activities | 10 weeks | 10 weeks | 4,9 |
| Harald et al.[63] | 2012 | Norway | Hypertension | 52.5 ±7.4 | 51.3 ±9.2 | 31(16/15) | 29(17/12) | mode of motion: running  exercise time: 16 mins  exercise frequency: three times per week  exercise intensity: 85-90% VO_2_max | mode of motion: daily activities | 12 weeks | 12 weeks | 4,7,8,10,11 |
| Han-Kyul et al[64] | 2017 | United States | Healthy | 65±1 | 63±2 | 17(4/13) | 14(4/10) | mode of motion: HIIT  exercise time: 25mins  exercise frequency: four times per week  exercise intensity: 90%HRmax | mode of motion: daily activities | 8 weeks | 8 weeks | 4,5,6,7,10,11,12,13 |
| Ha-Yoon et al.[65] | 2018 | Korea | Myocardial infarct | 53.00±6.84 | 57.31±12.62 | 23(21/2) | 21(18/3) | mode of motion: cycling  exercise time: 48 mins  exercise intensity: 85-100%HRmax | mode of motion: cycling  exercise time: 38 mins  exercise intensity: 60-70%HRmax | 9-10 weeks | 9-10 weeks | 4 |
| Han-Chow et al.[66] | 2018 | Denmark | Type 2 diabetes | 57±7 | 57±7 | 8(5/3) | 8(4/4) | mode of motion: cycling  exercise time: 20 mins  exercise frequency: three times per week  exercise intensity: 95% peak workload | mode of motion: cycling  exercise time: 40 mins  exercise frequency: three times per week  exercise intensity: 50% peak workload | 11 weeks | 11 weeks | 1,10,12,14 |
| Inès et al.[67] | 2019 | Canada | Healthy | 66.0 ± 3.4 | 64.2 ± 3.7 | 9(0/9) | 9(0/9) | mode of motion: running  exercise time: 16 mins  exercise frequency: three times per week  exercise intensity: 90% of HRR | mode of motion: moderate intensity aerobic exercise  exercise time: 45 mins  exercise frequency: three times per week  exercise intensity: 55% of HRR | 8 weeks | 8 weeks | 1,3,4,5,6,10,11,12,13 |
| James et al.[68] | 2016 | Australia | Cancer | 61.4±11.1 | 61.5±10.8 | 30 | 17 | mode of motion: cycling  exercise time: 16 mins  exercise frequency: three times per week  exercise intensity: 85–95 % HRmax | mode of motion: cycling  exercise time: 50 mins  exercise frequency: three times per week  exercise intensity: 70 %HRmax | 4 weeks | 4 weeks | 2,4 |
| Joachim et al.[69] | 2016 | Germany | Cancer | 53 ± 8 | 5 4 ± 9 | 13 | 13 | mode of motion: strenuous walking  exercise time: 8 mins  exercise frequency: three times per week  exercise intensity: >95% HRmax | mode of motion: initial incremental treadmill  exercise time: 75 mins  exercise frequency: two times per week  exercise intensity: 60% HRmax | 3 weeks | 3 weeks | 4 |
| Joyce et al[70] | 2016 | Australia | Metabolic syndrome | 57 ± 11 | 55±11 | 15 | 17 | mode of motion: treadmill  exercise time: 16 mins  exercise frequency: three times per week  exercise intensity: 85–95% HRmax | mode of motion: treadmill  exercise time: 30 mins  exercise frequency: five times per week  exercise intensity: 60–70% HRmax | 16 weeks | 16 weeks | 1,2,3,4,5,6,7,10,11,12,13 |
| Joyce et al.[71] | 2017 | Norway | Coronary heart disease | 56±8 | 54±11 | 19(9/10) | 16(9/7) | mode of motion: HIIT  exercise time: 25 mins  exercise frequency: three times per week  exercise intensity: 85-95% HRmax | mode of motion: MICT  exercise time: 17 mins  exercise frequency: five times per week  exercise intensity: 60-70%HRmax | 16 weeks | 16 weeks | 2,3,4,5,6,7 |
| James et al.[72] | 2018 | Australia | Cancer | 60.7 ± 11.7 | 59.8 ± 11.4 | 16 | 15 | mode of motion: cycling  exercise time: 38 mins  exercise frequency: four times per week  exercise intensity: 85-95%HRmax | mode of motion: cycling  exercise time: 50 mins  exercise frequency: three times per week  exercise intensity: 50-70%HRmax | 8 weeks | 8 weeks | 4 |
| Justin et al.[73] | 2018 | Australia | Osteoarthritis of knee join | 59.1±6.7 | 66.1±8.8 | 9(3/6) | 8(1/7) | mode of motion: cycling  exercise time: 13-14 mins  exercise frequency: four times per week  exercise intensity: at a rhythm of 70-110 rpm | mode of motion: cycling  exercise time: 20 mins  exercise frequency: four times per week  exercise intensity: at a rhythm of 60-80 rpm | 8 weeks | 8 weeks | 1,3 |
| Joseph et al.[74] | 2019 | Australia | Cancer | 60.3±8.1 | 61.5±7.8 | 6(0/6) | 6(0/6) | mode of motion: cycling  exercise time: 15-25 mins  exercise frequency: three times per week  exercise intensity: ＞90%HRmax | mode of motion: daily activities | 12 weeks | 12 weeks | 4 |
| Jose et al.[75] | 2019 | UK | Type 2 diabetes | 61.1±8.6 | 59.8±8.6 | 13(3/10) | 13(3/10) | mode of motion: ergometry sessions at a gym  exercise time:  exercise frequency: three times per week | mode of motion: daily activities | 12 weeks | 12 weeks | 5,6,7,8,9,  10,11,14 |
| Jenna et al.[76] | 2020 | Australia | coronary artery disease | 65±7 | 63±7 | 19 | 23 | mode of motion: HIIT  exercise time: 32 mins  exercise frequency: three times per week  exercise intensity: 85–95% HRmax | mode of motion: MICT  exercise time: 34 mins  exercise frequency: three times per week  exercise intensity: 65-75% HRmax | 4 weeks | 4 weeks | 1,3 |
| Jennifer et al.[77] | 2022 | Canada | Coronary artery disease | 61 ± 7 | 60 ± 7 | 43 | 44 | mode of motion: HIIT  exercise time: 16 mins  exercise frequency: two times per week  exercise intensity: 85–95% HRmax | mode of motion: MICT | 12 weeks | 12 weeks | 1,2,3,5,6 |
| Ken-Ichi et al.[78] | 2007 | Japan | Healthy | 67±4,64±6 | 66±5,62±6 | 42(11/31) | 46(9/37) | mode of motion: walk  exercise time: Three minutes each time  exercise frequency: ＞4days per week  exercise intensity: 70% VO_2_peak | mode of motion: sedentary lifestyle | 20 weeks | 20 weeks | 1,5,6,7,8 |
| Katharine et al.[79] | 2013 | Canada | Coronary heart disease | 62 ± 11 | 68 ± 8 | 11 | 10 | mode of motion: continuous cycling  exercise time: 30–50 mins  exercise frequency: two days per week  exercise intensity: 89% PPO | mode of motion: continuous cycling  exercise time: 30–50 mins  exercise frequency: two days per week  exercise intensity: 58%PPO | 12weeks | 12weeks | 4,5,6,7,8,9 |
| Katharine et al.[80] | 2013 | Canada | Coronary heart disease | 63±11 | 64±6 | 7(7/0) | 7(7/0) | mode of motion: continuous cycling  exercise time: 11 mins  exercise frequency: two days per week  exercise intensity: 88% PPO(range 80%–99%) | mode of motion: continuous cycling  exercise time: 30-50 mins  exercise frequency: two days per week  exercise intensity: 60% PPO (range 55%–65%) | 12weeks | 12weeks | 4,5,6,7,8,9 |
| Koldobika et al.[81] | 2013 | Spain | Coronary artery disease | 57.6 ± 9.8 | 58.3 ± 9.5 | 57 | 53 | mode of motion: training on a bicycle ergometer  exercise time: 40 mins  exercise frequency: three days per week | mode of motion: training on a bicycle ergometer  exercise time: 40 mins  exercise frequency: three days per week | 8 weeks | 8 weeks | 4,8,9 |
| Kellie et al.[82] | 2016 | Australia | Cancer | 51.6±13.01 | 51.6±13.01 | 8(0/8) | 8(0/8) | mode of motion: stationary bike or treadmill  exercise time: 24 mins  exercise frequency: three times per week  exercise intensity: ≥85%HRmax | mode of motion: stationary bike or treadmill  exercise time: 20 mins  exercise frequency: three times per week  exercise intensity: ≤55% HRmax | 12 weeks | 12 weeks | 2,3,5,6,7 |
| Koldobika et al.[83] | 2016 | Spain | Coronary heart disease | 58 ± 11 | 58 ± 11 | 36 | 36 | mode of motion: cycling  exercise time: 40 mins  exercise frequency: three days per week  exercise intensity: 104.5%±22.2%VO_2_max | mode of motion: cycling  exercise time: 40 mins  exercise frequency: three days per week  exercise intensity: 64.2±8.5% VO_2_max | 8 weeks | 8 weeks | 4,5,6,7,8,9 |
| Kamilla et al.[84] | 2018 | Denmark | Type 2 diabetes | 54±6 | 57±7 | 13(7/6) | 7(5/2) | mode of motion: cycling  exercise time: 20 mins  exercise frequency: three times per week  exercise intensity: 95% VO_2_max | mode of motion: daily activities | 11 weeks | 11 weeks | 1,4,5,6,7,  10,11,12,13,14 |
| Lianne et al.[85] | 2016 | Canada | Cancer | 56.2 ±9 | 59.4 ±9 | 12(0/12) | 10(0/10) | mode of motion: running  exercise time: 12-20 mins  exercise frequency: three times per week  exercise intensity: 80 % VO_2_max | mode of motion: daily activities | 6 weeks | 6 weeks | 3,4,7,14 |
| Liu et al.[86] | 2018 | China | Hypertension | 52.8±11.6 | 53.9±12.2 | 18(10/8) | 18(10/8) | mode of motion: running  exercise time: 20 mins  exercise frequency: three times per week  exercise intensity: 80%of HRR | mode of motion: running  exercise time: 20 mins  exercise frequency: three times per week  exercise intensity: 60%-65%of HRR | 16 weeks | 16 weeks | 5,6 |
| Lukas-Daniel et al.[87] | 2020 | Canada | Healthy | 63.6 ± 9.0 | 58.7 ± 11.3 | 23(15/8) | 9(7/2) | mode of motion: bicycle ergometer  exercise frequency: two-three times per week | mode of motion: daily activities | 12 weeks | 12 weeks | 8,9 |
| Markos et al.[88] | 2014 | UK | Healthy | 64±7 | 64±4 | 11(0/11) | 7(0/7) | mode of motion: HIIT  exercise time: 19 mins  exercise frequency: three times per week | mode of motion: continuous training  exercise time: 40 mins  exercise frequency: three times per week  exercise intensity: 65%PPO | 2weeks | 2weeks | 4,5,6,7 |
| Marc et al.[89] | 2017 | Switzerland | Cancer | 64±10 | 64±13 | 77(50/27) | 74(41/33) | mode of motion: HIIT  exercise time: 25 mins  exercise frequency: two-three times per week  exercise intensity: 80-100% WR peak | mode of motion: usual care | Unclear | Unclear | 4 |
| Mohammad et al.[90] | 2018 | Iran | Hypertension | 54.78 ± 6.19 | 54.24 ± 5.61 | 18(9/9) | 17(9/8) | mode of motion: cycling  exercise time: 43 mins  exercise frequency: three times per week  exercise intensity: 85%-90%HRmax, | mode of motion: daily activities | 12 weeks | 12 weeks | 1,4,5,6,8,  10,11,12,13,14 |
| Marcel et al.[91] | 2019 | Sweden | Healthy | 70.7±0.2 | 70.7±0.2 | 40(19/21) | 40(19/21) | mode of motion: severe intermittent exercise  exercise time: 18-36 mins  exercise frequency: three times per week | mode of motion: daily activities | 10 weeks | 10 weeks | 5,6,7,10,11,12,13 |
| Nele et al.[92] | 2016 | Belgium | Coronary heart disease | 57.4 ± 8.7 | 59.9 ± 9.2 | 80(76/4) | 83(76/7) | mode of motion: cycling  exercise time: 16 mins  exercise frequency: three times per week  exercise intensity: 85–95% HRmax | mode of motion: cycling  exercise time: 47 mins  exercise frequency: three times per week  exercise intensity: 70–75%HRmax | 12 weeks | 12 weeks | 1,4,5,6,7,8,9,12,13,14 |
| Nele et al.[93] | 2017 | Belgium | Coronary heart disease | 57.0 ± 8.8 | 59.9 ± 9.2 | 100(91/9) | 100(89/11) | mode of motion: AIT  exercise time: 25 mins  exercise frequency: three times per week  exercise intensity: 88% HRmax | mode of motion: ACT  exercise time: 37 mins  exercise frequency: three times per week  exercise intensity: 80% HRmax | 12 weeks | 12 weeks | 4,8 |
| NurAzis et al.[94] | 2023 | Indonesia | Healthy | 54.3±5.1 | 53.6±6.1 | 12 | 12 | mode of motion: cycling  exercise time: 38 mins  exercise frequency: three times per week  exercise intensity: 90-95% HRmax | mode of motion: cycling  exercise time: 47 mins  exercise frequency: three times per week  exercise intensity: 70-75% HRmax | 16 weeks | 16 weeks | 2,3,4,10,11,12,13,14 |
| Øivind et al.[95] | 2004 | Norway | Coronary heart disease | 62.9±11.2 | 61.2±7 .3 | 8(6/2) | 9(8/1) | mode of motion: treadmill walking  exercise time: 33mins exercise frequency: three times per week  exercise intensity: 80–90% VO_2_max (85–95% HRmax) | mode of motion: treadmill walking  exercise time: 41mins  exercise frequency: three times per week  exercise intensity：50–60%VO_2_max | 10 weeks | 10 weeks | 4,5,6,7,8,9 |
| Paolo et al.[96] | 2020 | Italy | Healthy | 69.4 ± 4.3 | 69.67 ± 4.1 | 12 | 12 | mode of motion: cycling exercise  exercise time: 14 mins  exercise frequency: three times per week  exercise intensity: 85-95%VO_2_max | mode of motion: moderate-intensity training  exercise time: 20-30 mins  exercise frequency: three times per week | 8 weeks | 8 weeks | 2,3,4 |
| Philip et al.[97] | 2020 | China | Coronary artery disease | 69±3 | 73±6 | 10(5/5) | 10(6/4) | mode of motion: HIIT  exercise time: 16.5 mins  exercise frequency: three times per week  exercise intensity: 90%-110%PPO | mode of motion: daily activities | 6 weeks | 6 weeks | 4 |
| P. Eser et al.[98] | 2022 | Switzerland | Myocardial infarct | 53 (49–66) | 59 (52–62) | 34 | 35 | mode of motion: cycling  exercise time: 16 mins  exercise intensity: borg score 15 to16 | mode of motion: cycling  exercise time: 38 mins  exercise frequency: three times per week  exercise intensity: borg score 13 to14 | 9 weeks | 9 weeks | 4,5,6,8 |
| Rikke et al.[99] | 2019 | Denmark | Lacunar stroke | 63.7 ± 8.9 | 63.7 ± 9.2 | 74(23/51) | 81(26/55) | mode of motion: cycling and walking  exercise time: 15 mins  exercise frequency: five times per week  exercise intensity: 77-93% HRmax | mode of motion: intensive exercise | 12 weeks | 12 weeks | 1,5,6,10,11,12,13 |
| Siri et al.[100] | 2014 | Norway | Type 2 diabetes | 58.6±5 | 54.7±5.3 | 24(15/9) | 23(5/8) | mode of motion: HIE  exercise time: 16 mins  exercise frequency: three times per week  exercise intensity: 90-95% HRmax | mode of motion: MIE  exercise time: 10 mins  exercise frequency: 210 mins per week | 12weeks | 12weeks | 1,2,3,4,5,6,7 |
| Steven et al.[101] | 2014 | America | Coronary heart disease | 60 ± 7 | 58 ± 9 | 21(11/10) | 18(12/6) | mode of motion: HIIT  exercise time: 25 mins  exercise intensity: 80-90% of HRR | mode of motion: MICT  exercise time: 30 mins  exercise intensity: 60-80% of HRR | 10 weeks | 10 weeks | 5,6,8,9 |
| Siddhartha et al.[102] | 2015 | United States | Heart failure | 69.0 ± 6.1 | 71.5 ± 11.7 | 9(8/1) | 6(4/2) | mode of motion: cycling  exercise time: 16-34 mins  exercise frequency: three days per week  exercise intensity: 80-85%PHR | mode of motion: cycling  exercise time: 15-30 mins  exercise frequency: three days per week  exercise intensity: 60-70%PHR | 4weeks | 4weeks | 1,4,5,6,8,9 |
| Sophie et al.[103] | 2016 | UK | Type 2 diabetes | 61±9 | 59±9 | 12(10/2) | 11(8/3) | mode of motion: cycling  exercise time: 18-27mins  exercise frequency: three times per week | mode of motion: daily activities | 12 weeks | 12 weeks | 5,6,10,11,14 |
| Srijit et al.[104] | 2017 | UK | Cancer | 71.60 ± 6.80 | 72.5 ± 8.40 | 30(3/27) | 30(4/26) | mode of motion: HIIT  exercise time: 30 mins  exercise frequency: two times per week  exercise intensity: 70-85% HRmax | mode of motion: daily activities | 3-6weeks | 3-6weeks | 4,8,9 |
| Stephan et al.[105] | 2021 | Germany | Healthy | 64.5 ± 7.2 | 66 ± 4.7 | 58 (17/41) | 60(19/41) | mode of motion: cycle ergometers  exercise time: 7 mins  exercise frequency: three times per week  exercise intensity: 80–90% HRmax | mode of motion: daily activities | 12 weeks | 12 weeks | 4 |
| Simon et al.[106] | 2023 | Switzerland | Hypertension | 56 ±6 | 59 ±7 | 19(13/6) | 19(11/8) | mode of motion: HIIT  exercise time: 7 mins  exercise frequency: three times per week  exercise intensity: 80–95% HRmax | mode of motion: daily activities | 8 weeks | 8weeks | 1,2,4,11,12,13,14 |
| Trine et al.[107] | 2009 | Norway | Coronary artery disease | 60.2±6.9 | 62.0±7.6 | 28(24/4) | 31(24/7) | mode of motion: walk  exercise time: 25 mins  exercise frequency: five days per week  exercise intensity: 90%VO_2_max | mode of motion: walk  exercise time: 25 mins  exercise frequency: five days per week  exercise intensity: 70%VO_2_max | 4 weeks | 4 weeks | 4,7,8,9,10,  12,13 |
| Trine et al.[108] | 2011 | Norway | Myocardial infarct | 56.7± 10.4 | 57.3± 9.7 | 30(25/5) | 72(60/12) | mode of motion: walk  exercise time: 16 mins  exercise frequency: two times per week  exercise intensity: 90%（85%-95%）VO_2_max | mode of motion: aerobic exercise  exercise time: 60 mins  exercise frequency: two times per week | 12 weeks | 12 weeks | 4,7,9,10,12 |
| Trine et al.[109] | 2012 | Norway | Myocardial infarction | 56.7±10.4 | 57.7 ±9.3 | 30(25/5) | 59(49/10) | mode of motion: aerobic interval training  exercise time: 16 mins  exercise frequency: once per week  exercise intensity: 85-95% HRmax | mode of motion: aerobic interval training  exercise time: 35 mins  exercise frequency: once per week | 12 weeks | 12 weeks | 4,8,9,11,12 |
| Tasuku et al.[110] | 2013 | Canada | Type 2 diabetes | 62±3 | 63±5 | 7 | 8 | mode of motion: stationary cycling and treadmill walking  exercise time: 30 mins  exercise frequency: five days per week  exercise intensity: 100% VO_2_max | mode of motion: stationary cycling and treadmill walking  exercise time: 30 mins  exercise frequency: five days per week  exercise intensity: 40% VO_2_max | 12weeks | 12weeks | 1,2,3,4,10,  11,12,13,14 |
| Tor et al.[111] | 2020 | Norway | Cerebral  apoplexy | 57.6±9.2 | 58.7± 9.2 | 36 | 34 | mode of motion: running  exercise time: 38 mins  exercise frequency: three times per week  exercise intensity: 85-95%HRmax | mode of motion: daily activities | 8 weeks | 8 weeks | 4,5,6,8,10,11,12,13 |
| Trevor et al.[112] | 2023 | America | Healthy | 51.66 ± 7.88 | 48.57 ± 7.46 | 15(5/10) | 15(5/10) | mode of motion: cycling  exercise time: 10 mins  exercise frequency: three times per week  exercise intensity: 77%– 93% HRmax | mode of motion: sedentary | 4 weeks | 4 weeks | 1 |
| Ulrik et al.[113] | 2007 | Norway | Heart failure | 76.5±9 | 75.5±13 | 9(7/2) | 9(6/3) | mode of motion: treadmill  exercise time: 25 mins  exercise frequency: three times per week  exercise intensity: 95% HRmax | mode of motion: treadmill  exercise time: 47 mins  exercise frequency: once every 3 weeks  exercise intensity: 70% HRmax | 12 weeks | 12 weeks | 4,7,9 |
| Viviane et al.[114] | 2015 | Belgium | Coronary heart disease | 57.0 ± 8.8 | 59.9 ± 9.2 | 85 | 89 | mode of motion: cycling  exercise frequency: three times per week  exercise intensity: 90% HRmax | mode of motion: cycling  exercise frequency: three times per week  exercise intensity: 70-75%HRmax | 12weeks | 12weeks | 1,3,4,5,6,7,8,9,10,11,  12,13,14 |
| Victoria et al.[115] | 2017 | Australia | Healthy | 69.4±3.5 | 69.4±3.5 | 8(6/2) | 7(3/4) | mode of motion: IIT  exercise time: 28 mins  exercise frequency: three times per week  exercise intensity: 90-95% HRmax | mode of motion: daily activities | 12 weeks | 12 weeks | 4,8 |
| Victor et al.[116] | 2020 | Brazil | Healthy | 80.3±5.8 | 80.9±4.6 | 15 | 15 | mode of motion: HIIT  exercise time: 16 mins  exercise frequency: two times per week  exercise intensity: 85–95% HRmax | mode of motion: MIIT  exercise time: 16 mins  exercise frequency: two times per week  exercise intensity: 55–75% HRmax | 8 weeks | 8 weeks | 2,5,6,7 |
| W. Mitranun et al.[117] | 2014 | Thailand | Type 2 diabetes | 61.2 ± 2.8 | 60.9 ± 2.4 | 14(5/9) | 15(5/10) | mode of motion: aerobic interval training  exercise time: 10 mins  exercise frequency: three times per week  exercise intensity: 80-85% VO_2_max | mode of motion: sedentary | 12weeks | 12weeks | 1,2,5,6,7,10,11,12,13,14 |
| Wolfram et al.[118] | 2017 | Switzerland | Cancer | 64±10 | 64±13 | 77(50/27) | 74(41/33) | mode of motion: HIIT  exercise time: 25 mins  exercise intensity: WR peak | mode of motion: usual care | 3 weeks | 3 weeks | 4 |
| Xiu-Min et al.[119] | 2018 | China | Type 2 diabetes | 46-79 | 45-77 | 46(25/21) | 46(25/21) | mode of motion: cycling  exercise time: 7 mins  exercise frequency: four-ten times per week  exercise intensity: 70%-90%HRmax | mode of motion: daily activities | 12 weeks | 12 weeks | 14 |
| Xi et al.[120] | 2021 | China | Healthy | 64.9 ± 3.45 | 63.9 ± 3.95 | 10 | 9 | mode of motion: HIIT  exercise time: 21 mins  exercise frequency: three times per week  exercise intensity: 90% VO_2_max | mode of motion: daily activities | 12weeks | 12 weeks | 1,2,4 |

Notes: 1 BMI,2BF%,3WC,4VO2max,5SBP,6DBP,7HRrest,8HRmax,9RER,10FPG,11TC,12TG,13HDL,14LDL

BMI： body mass index, BF%：body fat percent, WC ：waist circumference,VO_2max_: maximum oxygen uptake, SBP ：systolic blood pressure, DBP： diastolic blood pressure, HR_rest_：resting heart rate, HR_max_：maximum heart rate, RER：respiratory exchange rate, TC ：total cholesterol, TG ：triglyceride, HDL： high-density lipoprotein, LDL： low-density lipoprotein, FPG： fasting plasma glucose.
