# Supplementary material for: Effects of high-intensity interval training on physical morphology, cardiopulmonary function, and metabolic indicators in older adults: a systematic review and meta-analysis
Source: Front Endocrinol (Lausanne). 2025 Mar 25;16:1526991. doi: 10.3389/fendo.2025.1526991 (PMC11975580; doi:10.3389/fendo.2025.1526991)

**Additional file 3** Risk of bias assessment of each outcome in the randomized controlled trials

| **Included studies** | **Year** | **Random sequence**  **generation** | **Allocation concealment** | **Blinding of participants and providers** | **Blinding of assessors** | **Incomplete outcome data** | **Selective reporting** | **Other bias** |
| --- | --- | --- | --- | --- | --- | --- | --- | --- |
| Arnt et al.[34] | 2008 | Low risk | Low risk | Low risk | Low risk | Low risk | Low risk | Unclear |
| Alireza et al.[35] | 2018 | Low risk | Low risk | High risk | Low risk | Low risk | Low risk | Unclear |
| Alireza et al.[36] | 2018 | Low risk | Low risk | Low risk | Low risk | Low risk | Low risk | Unclear |
| A.M. et al.[37] | 2018 | Low risk | Low risk | High risk | Low risk | Low risk | Low risk | Unclear |
| Arne et al.[38] | 2019 | Low risk | Low risk | Low risk | Low risk | Low risk | Low risk | Unclear |
| Brage et al.[39] | 2008 | Low risk | Low risk | Low risk | Low risk | Low risk | Low risk | Low risk |
| Bianca et al.[40] | 2020 | Low risk | Low risk | Low risk | Low risk | Low risk | Low risk | Unclear |
| Céline et al.[41] | 2012 | Low risk | Unclear | Low risk | Low risk | Low risk | Low risk | Low risk |
| Chueh-Lung et al.[41] | 2012 | Low risk | Low risk | Low risk | Low risk | Low risk | Low risk | Unclear |
| Chul et al.[43] | 2015 | Low risk | Low risk | Low risk | Low risk | Low risk | Low risk | Low risk |
| Chetna et al.[44] | 2019 | Low risk | Low risk | Low risk | Low risk | Low risk | Low risk | Low risk |
| Christopher et al.[45] | 2019 | Low risk | Low risk | Low risk | Low risk | Low risk | Low risk | Unclear |
| Chueh-Lung et al.[46] | 2019 | Low risk | Low risk | Low risk | Low risk | Low risk | Low risk | Unclear |
| Darren et al.[47] | 2005 | Low risk | Low risk | Low risk | Low risk | Low risk | Low risk | Unclear |
| D. F . J. et al.[48] | 2016 | Unclear | Unclear | High risk | Low risk | Low risk | Low risk | Unclear |
| D.M.L. et al.[49] | 2016 | Low risk | Low risk | Low risk | Low risk | Low risk | Low risk | Unclear |
| Ene´as et al.[50] | 2012 | Low risk | Low risk | Low risk | Low risk | Low risk | Low risk | Low risk |
| Erik et al.[51] | 2014 | Low risk | Low risk | Low risk | Low risk | Low risk | Low risk | Low risk |
| Edvardsen et al.[52] | 2015 | Low risk | Low risk | Low risk | Low risk | Low risk | Low risk | Low risk |
| Ebrahim et al.[53] | 2018 | Low risk | Low risk | High risk | Low risk | Low risk | Low risk | Unclear |
| Emma et al.[54] | 2023 | Low risk | Low risk | Low risk | Low risk | Low risk | Low risk | Low risk |
| Ferdinando et al.[55] | 2013 | Low risk | Low risk | Low risk | Low risk | Low risk | Low risk | Low risk |
| Francesco et al.[56] | 2013 | Low risk | Low risk | Low risk | Low risk | Low risk | Low risk | Low risk |
| Ferdinando et al.[57] | 2014 | Low risk | Low risk | Low risk | Unclear | Low risk | Low risk | Low risk |
| Fang et al.[58] | 2020 | Low risk | Low risk | Low risk | Unclear | Low risk | Low risk | Unclear |
| Ferdinando et al.[59] | 2021 | Low risk | Low risk | High risk | Low risk | Low risk | Low risk | Unclear |
| Gao et al.[60] | 2015 | Low risk | Low risk | Low risk | Low risk | Low risk | Low risk | Low risk |
| Gustavo et al.[61] | 2015 | Low risk | Low risk | Low risk | Low risk | Low risk | Low risk | Low risk |
| Håvard et al.[62] | 2005 | Low risk | Low risk | Low risk | Low risk | Low risk | Low risk | Unclear |
| Harald et al.[63] | 2012 | Low risk | Low risk | Low risk | Low risk | Low risk | Low risk | Unclear |
| Han-Kyul et al.[64] | 2017 | Low risk | Low risk | Low risk | Unclear | Low risk | Low risk | Low risk |
| Ha-Yoon et al.[65] | 2018 | Low risk | Low risk | Low risk | Low risk | Low risk | Low risk | Unclear |
| Han-Chow et al.[66] | 2018 | Low risk | Low risk | Low risk | Low risk | Low risk | Low risk | Low risk |
| Inès et al.[67] | 2019 | Low risk | Low risk | Low risk | Low risk | Unclear | Low risk | Unclear |
| James et al.[68] | 2016 | Low risk | Low risk | Low risk | Low risk | Low risk | Low risk | Unclear |
| Joachim et al.[69] | 2016 | Unclear | Unclear | Low risk | Low risk | Low risk | Low risk | Unclear |
| Joyce et al.[70] | 2016 | Low risk | Unclear | Low risk | Low risk | Low risk | Low risk | Unclear |
| Joyce et al.[71] | 2017 | Low risk | Low risk | Low risk | Unclear | Low risk | Low risk | Low risk |
| James et al.[72] | 2018 | Low risk | Low risk | High risk | Low risk | Low risk | Low risk | Unclear |
| Justin et al.[73] | 2018 | Low risk | Low risk | Low risk | Low risk | Low risk | Low risk | Unclear |
| Joseph et al.[74] | 2019 | Low risk | Low risk | Low risk | Low risk | Low risk | Low risk | Unclear |
| Jose et al.[75] | 2019 | Low risk | Low risk | Low risk | Low risk | Low risk | Low risk | Unclear |
| Jenna et al.[76] | 2020 | Low risk | Low risk | Low risk | Low risk | Low risk | Low risk | Low risk |
| Jennifer et al.[77] | 2022 | Low risk | Low risk | High risk | Low risk | Low risk | Low risk | Unclear |
| Ken-Ichi et al.[78] | 2007 | Low risk | Low risk | Low risk | Low risk | Low risk | Low risk | Unclear |
| Katharine et al.[79] | 2013 | Low risk | Low risk | Low risk | Low risk | Low risk | Low risk | Low risk |
| Katharine et al.[80] | 2013 | Low risk | Low risk | Low risk | Low risk | Low risk | Low risk | Low risk |
| Koldobika et al.[81] | 2013 | Low risk | Low risk | Low risk | Low risk | Low risk | Low risk | Low risk |
| Kellie et al.[82] | 2016 | Unclear | Unclear | Low risk | Low risk | Low risk | Low risk | Unclear |
| Koldobika et al.[83] | 2016 | Low risk | Low risk | Low risk | Low risk | Low risk | Low risk | Unclear |
| Kamilla et al.[84] | 2018 | Low risk | Low risk | Low risk | Low risk | Low risk | Low risk | Unclear |
| Lianne et al.[85] | 2016 | Low risk | Low risk | Low risk | Low risk | Low risk | Low risk | Unclear |
| Liu et al.[86] | 2018 | Low risk | Unclear | Low risk | Low risk | Low risk | Low risk | Low risk |
| Lukas-Daniel et al.[87] | 2020 | Low risk | Low risk | Low risk | Low risk | Low risk | Low risk | Unclear |
| Markos et al.[88] | 2014 | Low risk | Low risk | Low risk | Unclear | Low risk | Low risk | Low risk |
| Marc et al.[89] | 2017 | Low risk | Low risk | Low risk | Low risk | Low risk | Low risk | Low risk |
| Mohammad et al.[90] | 2018 | Low risk | Low risk | Low risk | Low risk | Low risk | Low risk | Unclear |
| Marcel et al.[91] | 2019 | Low risk | Low risk | Low risk | Low risk | Low risk | Low risk | Unclear |
| Nele et al.[92] | 2016 | Low risk | Low risk | High risk | Low risk | Low risk | Low risk | Low risk |
| Nele et al.[93] | 2017 | Low risk | Low risk | Low risk | Unclear | Low risk | Low risk | Low risk |
| NurAzis et al.[94] | 2023 | Low risk | Low risk | Low risk | Low risk | Low risk | Low risk | Low risk |
| Øivind et al.[95] | 2004 | Low risk | Low risk | Low risk | Low risk | Low risk | Low risk | Unclear |
| Paolo et al.[96] | 2020 | Low risk | Low risk | Low risk | Low risk | Low risk | High risk | Unclear |
| Philip et al.[97] | 2020 | Low risk | Low risk | Low risk | Low risk | Low risk | Low risk | Unclear |
| P. Eser et al.[98] | 2022 | Low risk | Low risk | High risk | Low risk | Low risk | Low risk | Unclear |
| Rikke et al.[99] | 2019 | Low risk | Low risk | Low risk | Low risk | Low risk | Low risk | Unclear |
| Siri et al.[100] | 2014 | Low risk | Low risk | Unclear | Unclear | Low risk | Low risk | High risk |
| Steven et al.[101] | 2014 | Low risk | Low risk | Low risk | Unclear | Low risk | Low risk | Low risk |
| Siddhartha et al.[102] | 2015 | Low risk | Low risk | Low risk | Low risk | Low risk | Low risk | Low risk |
| Sophie et al.[103] | 2016 | Low risk | Low risk | Low risk | Low risk | Low risk | Low risk | Low risk |
| Srijit et al.[104] | 2017 | Low risk | Low risk | Low risk | Unclear | Low risk | Low risk | Low risk |
| Stephan et al.[105] | 2021 | Low risk | Low risk | High risk | Low risk | Low risk | Low risk | Unclear |
| Simon et al.[106] | 2023 | Low risk | Low risk | Low risk | Low risk | Low risk | Low risk | Low risk |
| Trine et al.[107] | 2009 | Low risk | Unclear | Low risk | Low risk | Low risk | Low risk | Low risk |
| Trine et al.[108] | 2011 | Low risk | Low risk | Low risk | Low risk | Low risk | Low risk | Low risk |
| Trine et al.[109] | 2012 | Low risk | Low risk | Low risk | Low risk | Low risk | Low risk | Unclear |
| Tasuku et al.[110] | 2013 | Low risk | Low risk | High risk | Low risk | Low risk | Low risk | Low risk |
| Tor et al.[111] | 2020 | Low risk | Low risk | Low risk | Low risk | Low risk | Low risk | Low risk |
| Trevor et al.[112] | 2023 | Low risk | Low risk | Low risk | Low risk | Low risk | Low risk | Low risk |
| Ulrik et al.[113] | 2007 | Low risk | Low risk | Low risk | Low risk | Low risk | Low risk | Unclear |
| Viviane et al.[114] | 2015 | Low risk | Low risk | High risk | Low risk | Low risk | Low risk | Unclear |
| Victoria et al.[115] | 2017 | Low risk | Low risk | Low risk | Low risk | Low risk | Low risk | Unclear |
| Victor et al.[116] | 2020 | Low risk | Low risk | High risk | Low risk | Low risk | Low risk | Unclear |
| W. Mitranun et al.[117] | 2014 | Low risk | Low risk | Low risk | Unclear | Low risk | Low risk | Low risk |
| Wolfram et al.[118] | 2017 | Low risk | Low risk | Low risk | Low risk | Low risk | Low risk | Unclear |
| Xiu-Min et al.[119] | 2018 | Low risk | Low risk | Low risk | Low risk | Low risk | Low risk | Unclear |
| Xi et al.[120] | 2021 | Low risk | Low risk | Low risk | Low risk | Low risk | Low risk | Low risk |

Analysis of the risk of bias according to the Cochrane collaboration guideline


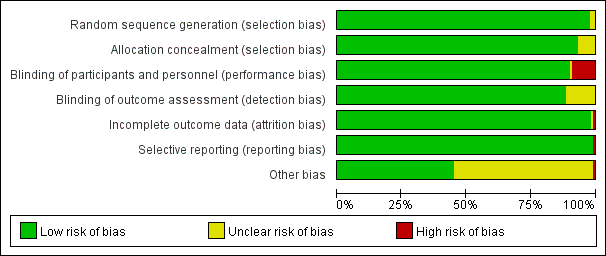

Supplement: Supplementary file 3 [file Table3.docx]
