# Supplementary material for: Effects of high-intensity interval training on physical morphology, cardiopulmonary function, and metabolic indicators in older adults: a systematic review and meta-analysis
Source: Front Endocrinol (Lausanne). 2025 Mar 25;16:1526991. doi: 10.3389/fendo.2025.1526991 (PMC11975580; doi:10.3389/fendo.2025.1526991)
Supplement: Supplementary file 4 [file Table4.docx]

**Fig funnel plot of BMI.**

**
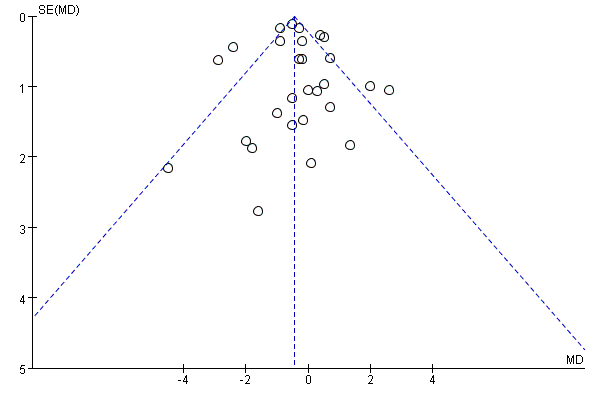
**

**Table subgroup analysis results of BF%.**

| Outcomes | Subgroup |  | The Num of studies | MD[95%CI] | I²（%） | P value | P Test for subgroup differences |
| --- | --- | --- | --- | --- | --- | --- | --- |
|  |  |  |  |  |  |  |  |
| BF% | Training volume (weeks) | ≤12weeks | 13 | -2.00 [-3.20, -0.81] | 32 | 0.001** | 0.07 |
|  |  | ＞12weeks | 3 | 1.03 [-2.00, 4.05] | 0 | 0.51 |  |
|  | Research object | ill | 12 | -1.80 [-3.19, -0.40] | 44 | 0.01* | 0.40 |
|  |  | health | 4 | -0.58 [-3.03, 1.86] | 0 | 0.64 |  |
|  | Disease | cardiovascular disease | 1 | -1.60 [-4.28, 1.08] | - | 0.24 | 0.55 |
|  |  | diabetes mellitus | 5 | -2.13 [-4.28, 0.01] | 68 | 0.05 |  |
|  |  | hypertension | 1 | -2.30 [-6.65, 2.05] | - | 0.30 |  |
|  |  | metabolic syndrome | 2 | 1.43 [-2.19, 5.06] | 0 | 0.44 |  |
|  |  | cancer | 2 | -1.95 [-8.89, 5.00] | 55 | 0.58 |  |
|  |  | Other diseases | 1 | -5.20 [-12.52, 2.12] | - | 0.16 |  |

*P＜0.05，**P＜0.01

**Fig funnel plot of BF%.**

**
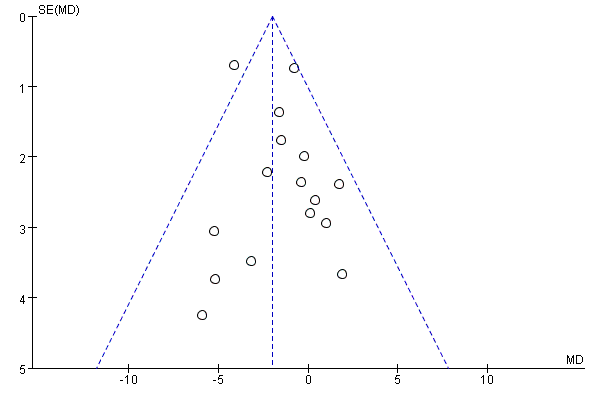
**

**Fig funnel plot of WC.**

**
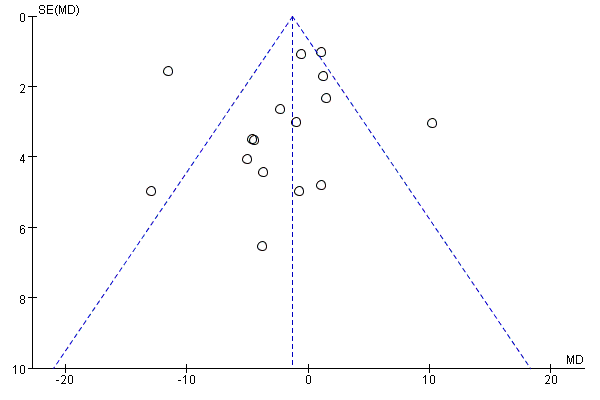
**

**Table subgroup analysis results of VO_2_max.**

| Outcomes | Subgroup |  | The Num of studies | MD[95%CI] | I²（%） | P value | P Test for subgroup differences |
| --- | --- | --- | --- | --- | --- | --- | --- |
|  |  |  |  |  |  |  |  |
| VO_2_max | Training volume (weeks) | ≤12weeks | 47 | 2.32 [1.60, 3.05] | 75 | < 0.00001** | 0.77 |
|  |  | ＞12weeks | 8 | 2.85 [-0.65, 6.35] | 90 | 0.11 |  |
|  | Research object | ill | 43 | 2.68 [1.78, 3.58] | 82 | < 0.00001** | 0.23 |
|  |  | health | 12 | 1.79 [0.66, 2.93] | 58 | 0.002** |  |
|  | Disease | cardiovascular disease | 22 | 1.93 [0.86, 3.01] | 78 | 0.0004** | 0.17 |
|  |  | diabetes mellitus | 5 | 4.40 [2.76, 6.04] | 34 | < 0.00001** |  |
|  |  | hypertension | 3 | 3.62 [1.10, 6.14] | 0 | 0.005** |  |
|  |  | metabolic syndrome | 3 | 3.87 [-5.22, 12.95] | 92 | 0.40 |  |
|  |  | cancer | 8 | 2.75 [1.42, 4.09] | 32 | ＜0.0001** |  |
|  |  | Other diseases | 2 | 1.48 [-0.73, 3.68] | 0 | 0.19 |  |

*P＜0.05，**P＜0.01

**Fig funnel plot of of VO_2_max.**

**
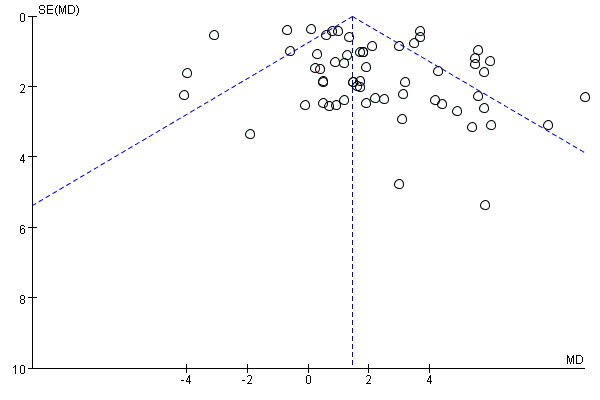
**

**Fig funnel plot of SBP.**

**
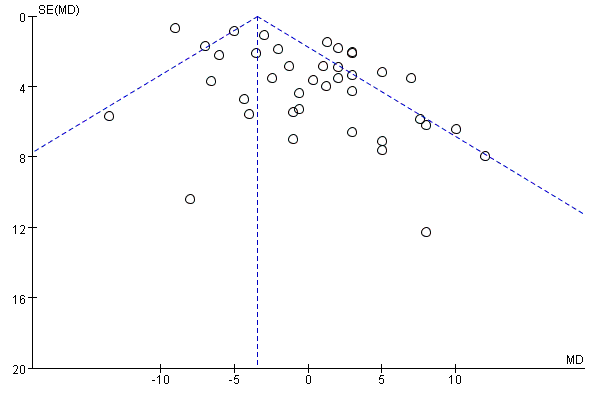
**

**Fig funnel plot of DBP.**

**
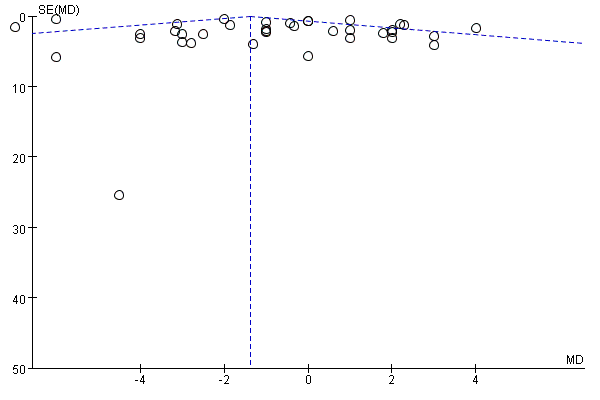
**

**Fig funnel plot of HRrest.**

**
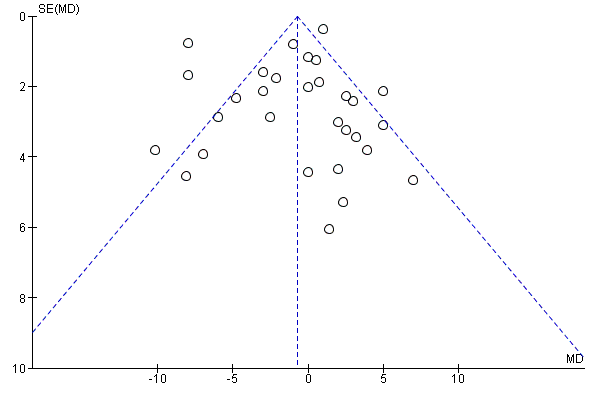
**

**Table subgroup analysis results of HRmax.**

| Outcomes | Subgroup |  | The Num of studies | MD[95%CI] | I²（%） | P value | P Test for subgroup differences |
| --- | --- | --- | --- | --- | --- | --- | --- |
|  |  |  |  |  |  |  |  |
| HRmax | Training volume (weeks) | ≤12weeks | 33 | 3.56 [1.87,5.25] | 33 | <0.0001** | 0.32 |
|  |  | ＞12weeks | 6 | 0.85[-4.43,6.12] | 83 | 0.84 |  |
|  | Research object | ill | 34 | 3.08 [0.91,5.25] | 59 | 0.005** | 0.65 |
|  |  | health | 5 | 1.55 [-4.72,7.81] | 87 | 0.63 |  |
|  | Disease | cardiovascular disease | 23 | 4.71[2.49,6.94] | 28 | <0.0001** | <0.0001** |
|  |  | diabetes mellitus | 3 | 3.67[1.21,6.13] | 0 | 0.003** |  |
|  |  | hypertension | 2 | 1.04[-6.54,8.63] | 59 | 0.79 |  |
|  |  | metabolic syndrome | 2 | -5.92[-9.40,-2.43] | 0 | 0.0009** |  |
|  |  | cancer | 3 | 5.30[-5.27,15.88] | 60 | 0.33 |  |
|  |  | Other diseases | 1 | -3.08[-10.18,4.02] | - | 0.40 |  |

*P＜0.05，**P＜0.01

**Fig funnel plot of HRmax.**

**
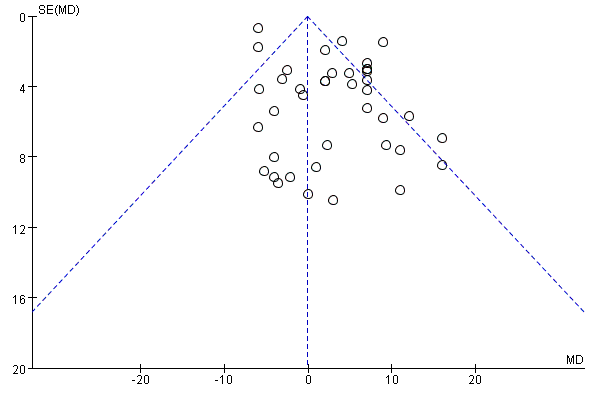
**

**Fig funnel plot of RER.**

**
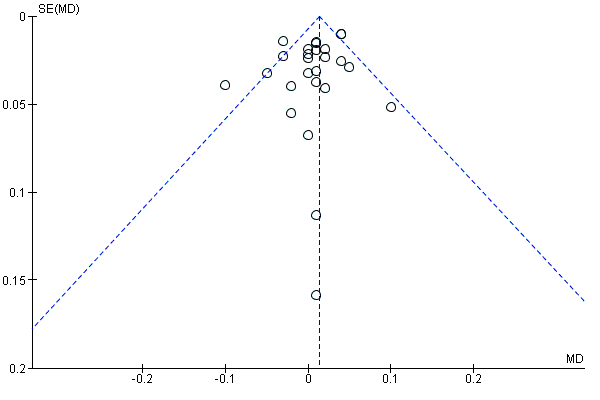
**

**Fig funnel plot of TC.**

**
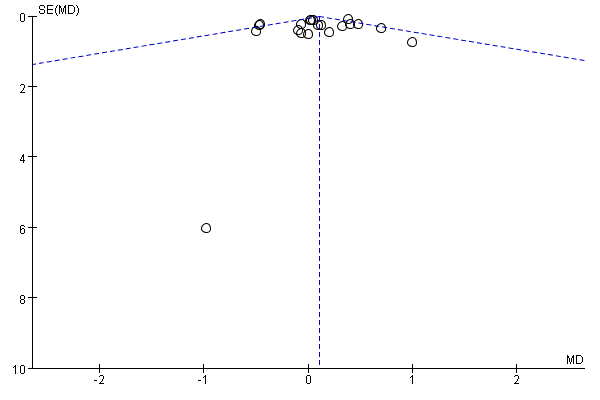
**

**Fig funnel plot of TG.**

**
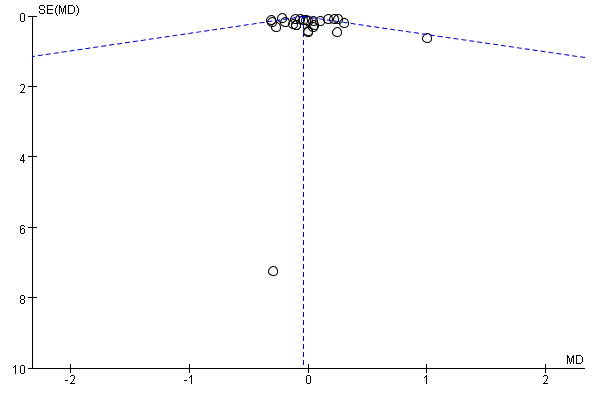
**

**Table subgroup analysis results of HDL.**

| Outcomes | Subgroup |  | The Num of studies | MD[95%CI] | I²（%） | P value | P Test for subgroup differences |
| --- | --- | --- | --- | --- | --- | --- | --- |
|  |  |  |  |  |  |  |  |
| HDL | Training volume (weeks) | ≤12weeks | 23 | 0.04 [0.01, 0.08] | 28 | 0.02* | 0.65 |
|  |  | ＞12weeks | 2 | -0.01 [-0.22, 0.21] | 0 | 0.95 |  |
|  | Research object | ill | 20 | 0.05 [0.01, 0.08] | 14 | 0.006** | 0.45 |
|  |  | health | 5 | -0.00 [-0.13, 0.12] | 48 | 1.00 |  |
|  | Disease | cardiovascular disease | 9 | 0.03 [-0.01, 0.08] | 6 | 0.13 | 0.46 |
|  |  | diabetes mellitus | 6 | 0.12 [0.01, 0.22] | 40 | 0.03* |  |
|  |  | hypertension | 1 | -0.09 [-0.30, 0.12] | - | 0.41 |  |
|  |  | metabolic syndrome | 1 | -0.01 [-0.28, 0.26] | - | 0.94 |  |
|  |  | cancer | 0 | - | - | - |  |
|  |  | Other diseases | 3 | 0.05 [-0.02, 0.13] | 0 | 0.16 |  |

*P＜0.05，**P＜0.01

**Fig funnel plot of HDL.**

**
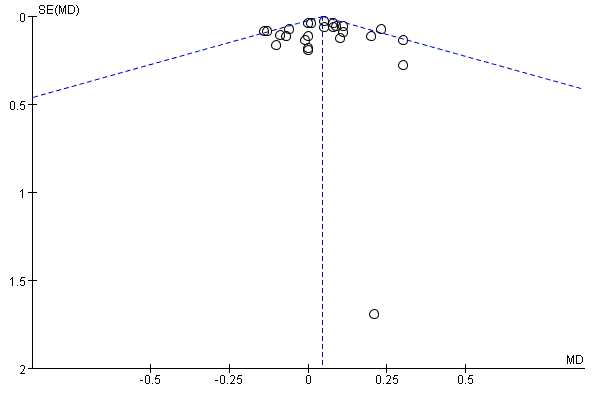
**

**Fig funnel plot of LDL.**

**
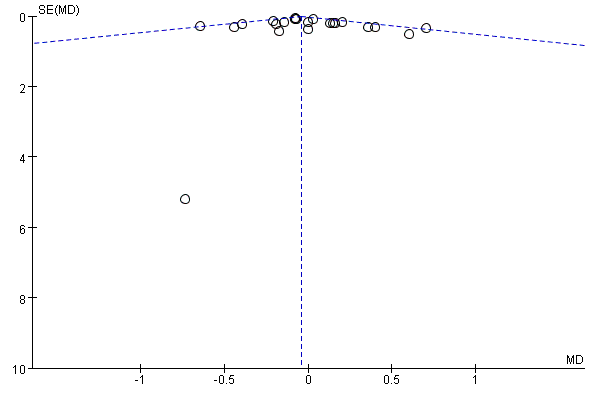
**

**Fig funnel plot of FPG.**

**
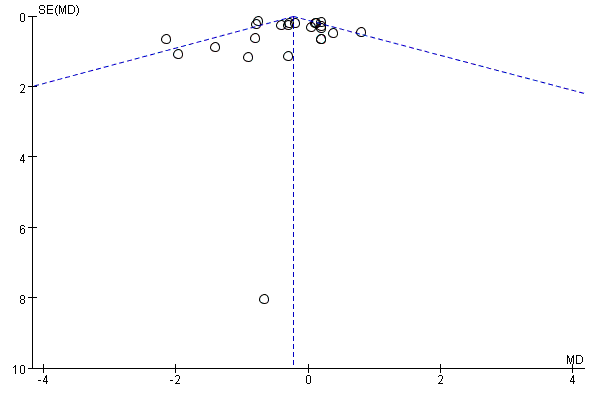
**
